# Supplementary material for: Quantifying the role of contact sampling for poliovirus detection in Nigeria
Source: PLOS Glob Public Health. 2026 May 13;6(5):e0006371. doi: 10.1371/journal.pgph.0006371 (PMC13170847; doi:10.1371/journal.pgph.0006371)
Supplement: S1 Text — (DOCX) [file pgph.0006371.s001.docx]

# Supplementary method

**Machine learning models’ framework**

We identified factors associated with the probability of finding i) a VDPV2 contact, or ii) a False Negative AFP through fitting Boosted Regression Trees (BRT). This machine learning approach allows for non-linear relationships of variables in the model and maximises the explanation of the variance by each factor [1].

**How individual BRT models are built:**

At each step of the tree-building process (i.e. each node), the model chooses a factor’s threshold that best discriminates the data into two groups and thus maximises the explanation of the variance. Then, at the next step/node, the model will only try to address the residual variance and so on until the stopping criteria are reached. To select the best combinations at each step, the model divides the data into training and test sets, repeatedly resampling and testing the model. We refer to this repeated train-and-test process used to build the model as inner testing to better differentiate it from the testing process used across several models to build the ensemble model. Figure S1 presents the flow diagram of the ensemble models built for this analysis.

**How the ensemble model is built:**

The ensemble model was built by iterating through a series of BRT models. At each iteration, the data is resampled and divided into two sets:

- the fitting set, used to tune the hyperparameters, fit and inner-test the model; and,
- -the cross-validation (CV) set, used to test the performance of the model on data that haven’t been used to fit the model.

For each individual fitting dataset, a BRT model is built, the factors’ importance is assessed, random and spatial resampling are realised to inner-test the performance of the model. Predictions are then calculated on the full data set and simulated data to obtain the marginal effects. Finally, the model accuracy is assessed through comparing model predictions and observations using the CV dataset.

Hyperparameters define the characteristics of a machine learning model, how it learns and the criteria to stop the process. The optimal model for the data and underlying process described is obtained through tuning of some of those hyperparameters. Here, a nested resampling has been applied to tune the number of trees built by the model (values between 50 and 3000) and the interaction depth, i.e., the number of nodes of each tree (values between 1 and 5). The optimal values have been evaluated over 15 iterations using a grid search method.

Table a.: model hyperparameters and ranges tested through nested resampling.

| **Hyperparameter** | **Min value** | **Max value** | **Evaluation method** | **N evaluations** |
| --- | --- | --- | --- | --- |
| number of trees | 50 | 3000 | grid | 15 |
| interaction depth | 1 | 5 | grid | 15 |

The factors selected to build the ensemble model include AFP case characteristics (Age, sex and the number of days between the onset of paralysis and the first stool collection); as well as, factors related to the contact sampling (the number of contacts sampled and the delay between the AFP’s onset and the first contact stool collection); and, local epidemic characteristics (the number of True Positive VDPV2 AFP cases in the same state in the month before the stool collection of the contact and the relative timing of nOPV2 activities organised in the department, LGA level). A full description of the factors is available in Table S1. The contribution of each of those factors to the model was quantified by relative importance, i.e. the weight of each factor in the model. This measure combines the number of times a factor has been used and how much variance it has contributed to explain, and is thus relative to the data and other variables and does not have a unit. The association between the factors and the probability of VDPV2 contact or FN AFP have been explored using marginal effects which represent how the probability varies depending on the value taken by the factors under study while all other factors are kept equal.

All the statistical analyses were performed in RStudio 2023.06.0 [2] using R version R-4.3.1. [3] The BRT models have been built using the R-package mlr3 [4], the figures have been built using the R-package ggplot2 [5] and the maps have been realised using QGIS 3.34 Prizren [6]. The R-code used for the analyses will be available on GitHub upon publication.

**Modelling the probability for a VDPV2 AFP to have at least one cVDPV2 contact**

The data included in this analysis consisted of all the VDPV2 AFP cases having between 1 and 3 contacts, excluding the FN AFP. Among them, 42% (n=140/331) had VDPV2 contacts and 58% (n=191/331) had negative contacts. The model here was built over 100 iterations with a resampling strategy based on a fitting set made of 86% (n=120) of the AFP with VDPV2 contacts and 63% (n=120) of the AFP with negative contacts to maximise the balance of positive and negative in the fitting set. Observations with missing data on any factor will be ignored.

**Modelling the characteristics of the False Negative**

The data included in this analysis consisted of all the VDPV2 AFP that had between 1 and 3 contacts. Among them, 25% (n=109/440) were FN AFP and 75% (n=331/440) were TP AFP.

Again, a BRT ensemble model was used to assess the probability for a VDPV2 AFP to be False Negative. Ten iterations of the model have been run on subsets of the data following the process described in Figure S1. Here the fitting set is built with 95% (n=104/109) of the FN AFP and 98% (n=326/331) of the TP at each iteration.

The factors selected to build the ensemble model include the same factors as the VDPV2 contact model except for the sex of the AFP case and the time between the onset of AFP and the contacts’ stool collection. Those factors have been removed to simplify the model and improve the model’s performance. Also, the year has been added because the definition of the FN AFP and the testing recommendations have changed over time (Table S1). Observations with missing data on any factor will be ignored.

# References:

[1] Elith J, Leathwick JR, Hastie T. A working guide to boosted regression trees. J Anim Ecol 2008;77:802–13. https://doi.org/10.1111/j.1365-2656.2008.01390.x.

[2] R Studio. RStudio – RStudio n.d. https://www.rstudio.com/products/rstudio/ (accessed February 15, 2017).

[3] R Core Team. R: A language and environment for statistical computing 2020.

[4] Lang M, Binder M, Richter J, Schratz P, Pfisterer F, Coors S, et al. mlr3: A modern object-oriented machine learning framework in R. J Open Source Softw 2019;4:1903. https://doi.org/10.21105/joss.01903.

[5] Create Elegant Data Visualisations Using the Grammar of Graphics n.d. https://ggplot2.tidyverse.org/ (accessed July 24, 2024).

[6] QGIS.org, 2021. QGIS Geographic Information System n.d.
