# Supplementary material for: Quantifying the role of contact sampling for poliovirus detection in Nigeria
Source: PLOS Glob Public Health. 2026 May 13;6(5):e0006371. doi: 10.1371/journal.pgph.0006371 (PMC13170847; doi:10.1371/journal.pgph.0006371)
Supplement: S1 Table — The observations containing errors have either been fully removed from the dataset or the error has been recoded as NAs. In those instances, if the variable with missing information is used in a model, this observation will be excluded in the analysis. (DOCX) [file pgph.0006371.s002.docx]

**S1 Table: AFP data cleaning process (data from Nigeria 1^st^ Dec 2016 - 30^th^ Nov 2023).** The observations containing errors have either been fully removed from the dataset or the error has been recoded as NAs. In those instances, if the variable with missing information is used in a model, this observation will be excluded in the analysis.

| Error tested | Nb errors | Action to correct | Nb corrected | Nb remain |
| --- | --- | --- | --- | --- |
| Non-AFP identifier | 6 | Remove observations | 6 | 0 |
| Wrong surveillance type | 0 | Remove observations | 0 | 0 |
| Missing date of onset | 0 | Remove observations | 0 | 0 |
| Results pending and onset date> 6 months | 48 | NA | 0 | 48 |
| Missing date stool 1 and onset date> 6 months | 2 | NA | 0 | 2 |
| Missing date stool 2 and onset date> 6 months | 5 | NA | 0 | 5 |
| Stool collection before onset | 0 | NA | 0 | 0 |
| Stool1 collected more than 6 months after onset | 2 | NA | 0 | 2 |
