# Supplementary material for: Quantifying the role of contact sampling for poliovirus detection in Nigeria
Source: PLOS Glob Public Health. 2026 May 13;6(5):e0006371. doi: 10.1371/journal.pgph.0006371 (PMC13170847; doi:10.1371/journal.pgph.0006371)
Supplement: S2 Table — The observations containing errors have been either fully removed from the dataset, corrected based on other contacts (there are 3 contacts sampled per AFP case in general), or recoded as NAs. In those instances, if the variable with missing information is used in a model, this observation will be excluded. (DOCX) [file pgph.0006371.s003.docx]

**S2 Table: Contact data cleaning flowchart (Nigeria 1^st^ Jan 2017 - 30^th^ Nov 2023).** The observations containing errors have been either fully removed from the dataset, corrected based on other contacts (there are 3 contacts sampled per AFP cases in general), or recoded as NAs. In those instances, if the variable with missing information is used in a model, this observation will be excluded.

| Error tested | Nb errors | Action to correct | Nb corrected | Nb remain |
| --- | --- | --- | --- | --- |
| Duplicates | 203 | Remove observations | 203 | 0 |
| Non-contact identifier | 38 | Correct misspellings when possible | 9 | 29 |
| Contact not matched with an AFP based on identifiers | 166 | Correct misspellings when possible | 81 | 86 |
| Date of stool collection <2016 | 8 | Correct the date based on other contacts | 8 | 0 |
| Stools sent to the lab before stool collection | 4 | Correct the date based on other contacts | 4 | 0 |
| Stools sent to the lab more than 20 days after stool collection | 209 | Correct the date based on other contacts | 109 | 100 |
| Stools received by the lab before being sent | 0 | Correct the date based on other contacts | 0 | 0 |
| Samples that need more than 5 days to travel | 860 | NA | 0 | 860 |
| Samples received by the lab more than 300 days after being sent | 2 | Correct the date based on other contacts | 2 | 0 |
| Missing date of the onset of the index APF | 86 | NA | 0 | 86 |
| Samples collected before the onset of the index AFP | 178 | Correct the date based on other contacts if possible | 164 | 14 |
