# Supplementary material for: Quantifying the role of contact sampling for poliovirus detection in Nigeria
Source: PLOS Glob Public Health. 2026 May 13;6(5):e0006371. doi: 10.1371/journal.pgph.0006371 (PMC13170847; doi:10.1371/journal.pgph.0006371)
Supplement: S3 Table — The grey areas correspond to the factors used in each model. (DOCX) [file pgph.0006371.s004.docx]

**S3 Table: Description of the factors included in the models.** The grey areas correspond to the factors used in each model.

| **Factor name** | **type** | **unit** | **VDPV2 contact model (n=331)** | | **FN AFP model (n=440)** | |
| --- | --- | --- | --- | --- | --- | --- |
|  |  |  | **distribution** | | **distribution** | |
|  |  |  | **median (interquartile)** | **frequencies** | **median (interquartile)** | **frequencies** |
| number of sampled contacts per AFP index case | continuous | number of people | 3(3-3) |  | 3(3-3) |  |
| nOPV2 activities in the last 6 months | factor | never/within the last 6 months/ more than 6 months ago |  | never: 15%/within the last 6 months:0%/ more than 6 months ago:85% |  | never: 12%/within the last 6 months:0.5%/ more than 6 months ago:87.5% |
| sex of AFP case | factor | Female/Male |  | Female: 47%/Male:53% |  |  |
| delay between AFP case’s stool collection to contact’s stool collection | continuous | number of days | 1(0-1) |  | 1(0-1) |  |
| delay between AFP onset to AFP case’s stool collection | continuous | number of days | 8(5-10) |  | 8(6-10) |  |
| number of True Postive AFP cases in the last month | continuous | number of person | 3(1-9) |  | 3(1-8) |  |
| age of AFP case (months) | continuous | number of months | 26 (18-37) |  | 26(19-38) |  |
| delay between AFP onset and contact’s stool collection | continuous | number of days | 9(6-11) |  |  |  |
| year of paralysis onset | continuous | years |  |  | 2021 (2021-2021) |  |
