# Supplementary material for: Quantifying the role of contact sampling for poliovirus detection in Nigeria
Source: PLOS Glob Public Health. 2026 May 13;6(5):e0006371. doi: 10.1371/journal.pgph.0006371 (PMC13170847; doi:10.1371/journal.pgph.0006371)
Supplement: S4 Table — The surveillance type refers to the surveillance system through which the virus was detected. (DOCX) [file pgph.0006371.s005.docx]

**S4 Table: Stratification of the first 10 detections of each VDPV2 emergence group that has circulated in Nigeria between 2015 and 2023 by surveillance type.** The surveillance type refers to the surveillance system through which the virus was detected

| Emergence group | year of first detection | Total number of detections | surveillance type for the first 10 detections | | | |
| --- | --- | --- | --- | --- | --- | --- |
|  |  |  | AFP | contact | ES | other |
| NIE-SOS-2 | 2016 | 2 | 1 | 1 | 0 | 0 |
| NIE-JIS-1 | 2018 | 265 | 3 | 0 | 7 | 0 |
| NIE-SOS-3 | 2018 | 26 | 0 | 0 | 10 | 0 |
| NIE-SOS-7 | 2019 | 58 | 3 | 3 | 4 | 0 |
| NIE-KGS-1 | 2019 | 8 | 3 | 0 | 4 | 1 |
| NIE-SOS-6 | 2019 | 5 | 0 | 0 | 5 | 0 |
| NIE-KGS-2 | 2019 | 5 | 2 | 0 | 0 | 3 |
| NIE-SOS-5 | 2019 | 2 | 1 | 0 | 0 | 1 |
| NIE-SOS-4 | 2019 | 4 | 0 | 0 | 4 | 0 |
| NIE-ZAS-1 | 2020 | 1112 | 6 | 0 | 4 | 0 |
| NIE-SOS-8 | 2020 | 11 | 2 | 7 | 1 | 0 |
| NIE-KBS-1 | 2021 | 3 | 3 | 0 | 0 | 0 |
