# Supplementary material for: Quantifying the role of contact sampling for poliovirus detection in Nigeria
Source: PLOS Glob Public Health. 2026 May 13;6(5):e0006371. doi: 10.1371/journal.pgph.0006371 (PMC13170847; doi:10.1371/journal.pgph.0006371)
Supplement: S5 Table — (DOCX) [file pgph.0006371.s006.docx]

**S5 Table: Correspondence of poliovirus isolation by serotype in the stools of the AFP contacts compared with the virus isolation result from the stool samples of the AFP index case**

| **Polio viruses combination in contacts** | **Polio viruses combination in the index AFP case** | | | | | | | |  |  |
| --- | --- | --- | --- | --- | --- | --- | --- | --- | --- | --- |
|  | **Neg** | **V1** | **V2** | **V3** | **V1+V2** | **V1+V3** | **V2+V3** | **V1+V2+V3** | **total** | **% of concordant** |
| Neg | **45884** | 275 | 1106 | 513 | 1 | 381 | 18 | 13 | 48191 | 95% |
| V1 | 160 | **17** | 2 | 12 | **0** | **16** | 0 | **0** | 207 | 16% |
| V2 | 750 | 0 | **476** | 7 | **2** | 2 | **3** | **1** | 1241 | 39% |
| V3 | 335 | 17 | 7 | **71** | 0 | **35** | **0** | **0** | 465 | 23% |
| V1+V2 | 0 | **0** | **0** | 0 | **0** | **0** | **0** | **0** | 0 | 0% |
| V1+V3 | 167 | **21** | 1 | **27** | **0** | **61** | **0** | **0** | 277 | 39% |
| V2+V3 | 5 | 0 | **7** | **1** | **0** | **0** | **2** | **0** | 15 | 67% |
| V1+V2+V3 | 0 | **0** | **0** | **0** | **0** | **0** | **1** | **1** | 2 | 100% |
| total | 47301 | 330 | 1599 | 631 | 3 | 495 | 24 | 15 | 50398 |  |
| % of concordant | 97% | 12% | 30% | 16% | 67% | 23% | 25% | 13% |  |  |
