# Supplementary material for: Quantifying the role of contact sampling for poliovirus detection in Nigeria
Source: PLOS Glob Public Health. 2026 May 13;6(5):e0006371. doi: 10.1371/journal.pgph.0006371 (PMC13170847; doi:10.1371/journal.pgph.0006371)
Supplement: S6 Table — (DOCX) [file pgph.0006371.s007.docx]

**S6 Table: performances of the models averaged across model iterations and overall when using median predictions of each ensemble model**

| model | probability of finding a cVDPV2 contact | probability of false negative AFP |
| --- | --- | --- |
| *performances averaged across model iterations:* |  |  |
| fitting-set median classification error (IQ) | 0.45 (IQ=0.43-0.48) | 0.26 (0.25-0.28) |
| cross-validation set median classification error (IQ) | 0.46 (IQ=0.44-0.49) | 0.40 (0.40-0.50) |
| mean sensitivity (sd) | 0.74 (0.04) | 0.36 (0.26) |
| mean specificity (sd) | 0.66 (0.06) | 0.98 (0.01) |
| mean accuracy (sd) | 0.69 (0.04) | 0.83 (0.06) |
|  |  |  |
| *overall ensemble model performance:* |  |  |
| sensitivity | 0.81 | 0.3 |
| specificity | 0.71 | 0.99 |
| accuracy | 0.75 | 0.82 |
