# Supplementary material for: Quantifying the role of contact sampling for poliovirus detection in Nigeria
Source: PLOS Glob Public Health. 2026 May 13;6(5):e0006371. doi: 10.1371/journal.pgph.0006371 (PMC13170847; doi:10.1371/journal.pgph.0006371)
Supplement: S7 Table — (DOCX) [file pgph.0006371.s008.docx]

**Importance of timeliness in AFP and contact stool sampling**

**S7 Table: Timeliness of AFP and contacts' stool collection**

|  | **AFP stools collected** | |  | **AFP having contact** | |
| --- | --- | --- | --- | --- | --- |
|  | **n** | **%** |  | **n** | **%** |
| **2017-2023:** |  |  |  |  |  |
| total | 71002 | 1 |  | 17917 | 1 |
| within 5 days | 23842 | 0.336 |  | 4999 | 0.279 |
| 6 to 15 days after onset | 44909 | 0.633 |  | 11104 | 0.620 |
| more than 15 days after onset | 2166 | 0.031 |  | 1796 | 0.100 |
|  |  |  |  |  |  |
| **2023 only:** |  |  |  |  |  |
| total | 11333 |  |  | 3745 |  |
| within 5 days | 4204 | 0.371 |  | 1239 | 0.331 |
| 6 to 15 days after onset | 6877 | 0.607 |  | 2338 | 0.624 |
| more than 15 days after onset | 246 | 0.022 |  | 168 | 0.045 |
