# Supplementary figures and images for: Quantifying the role of contact sampling for poliovirus detection in Nigeria
Source: PLOS Glob Public Health. 2026 May 13;6(5):e0006371. doi: 10.1371/journal.pgph.0006371 (PMC13170847; doi:10.1371/journal.pgph.0006371)

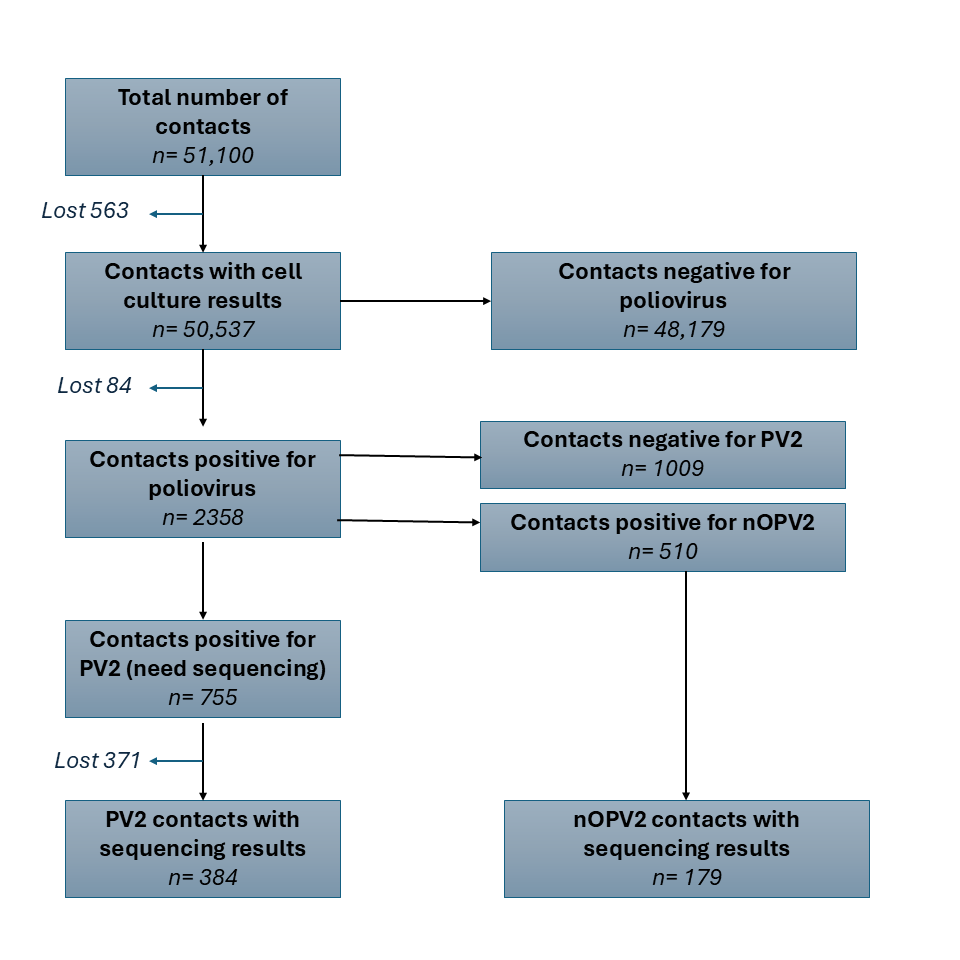

Supplement: S1 Fig — This can either be missing data entries or samples that have not been fully tested. (TIF) [file pgph.0006371.s009.tif]

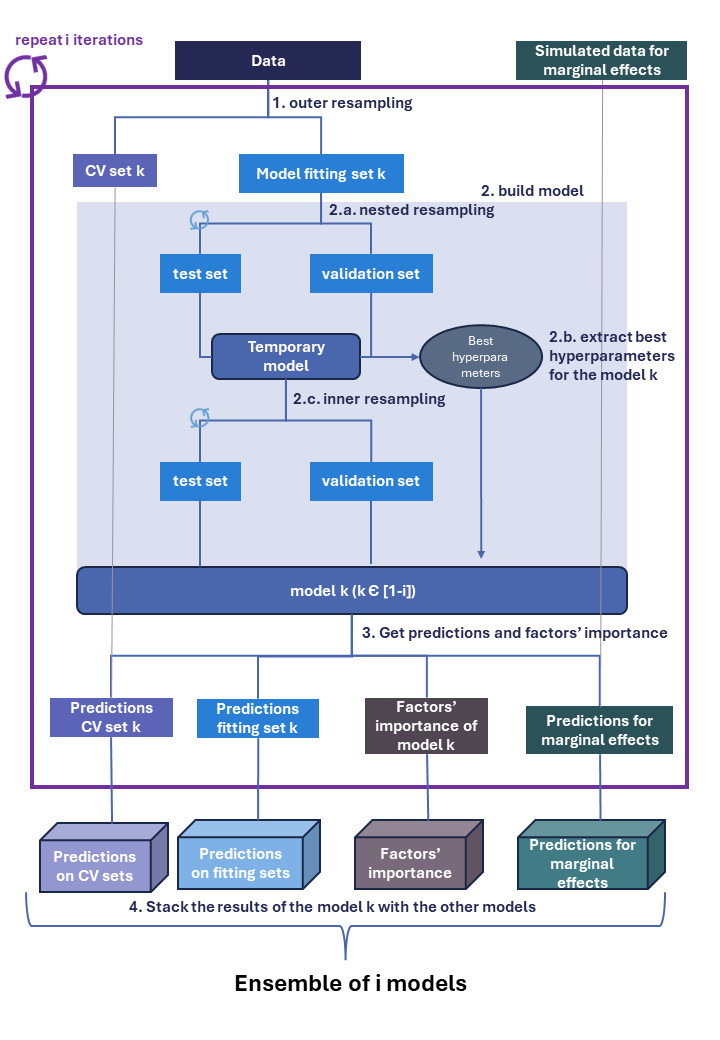

Supplement: S2 Fig — (TIF) [file pgph.0006371.s010.tif]

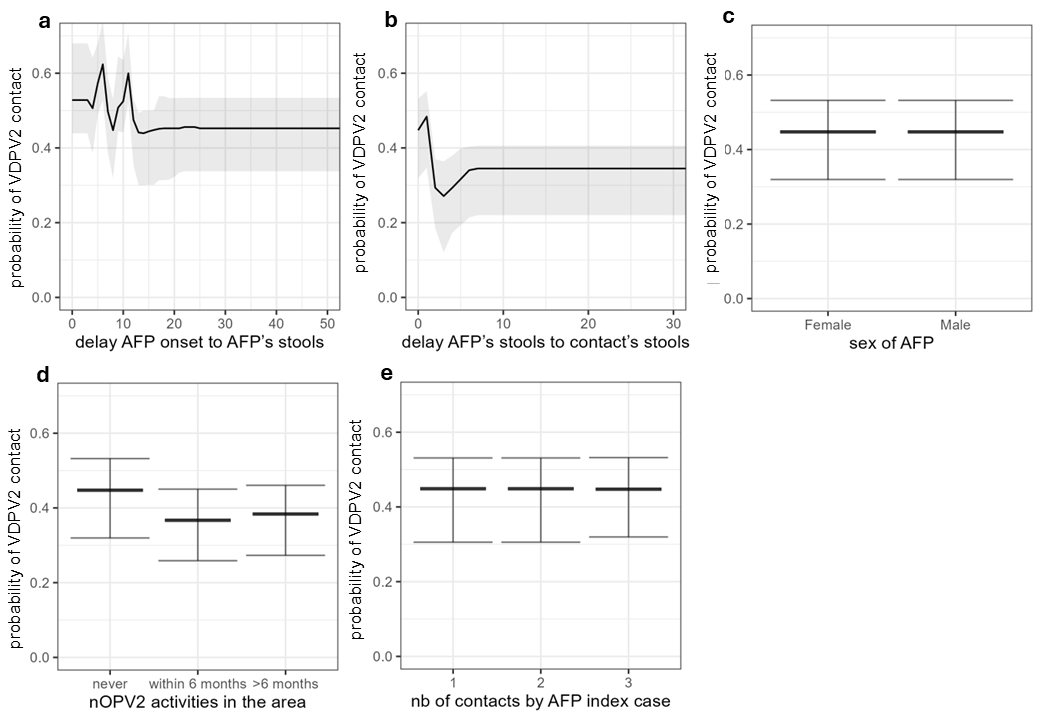

Supplement: S3 Fig — a. delay between AFP onset and AFP case’s stool collection; b. delay between AFP case’s stool collection and contacts’ stool collection; c. sex of the AFP case; d. nOPV2 activities conducted in the state; e. number of contact samples per AFP case. (TIF) [file pgph.0006371.s011.tif]

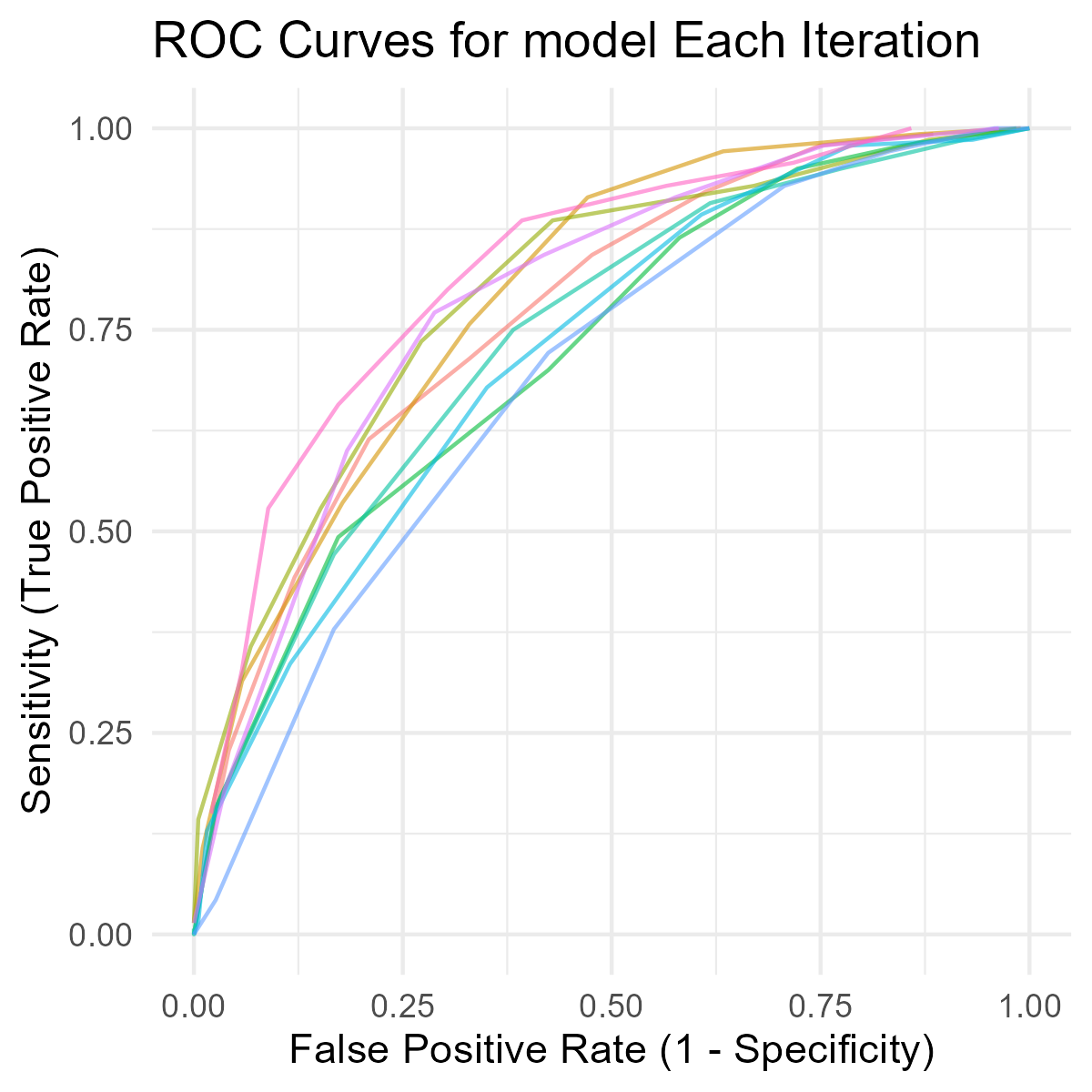

Supplement: S4 Fig — The 10 curves represented correspond to the first 10 iterations of the model. (PNG) [file pgph.0006371.s012.png]

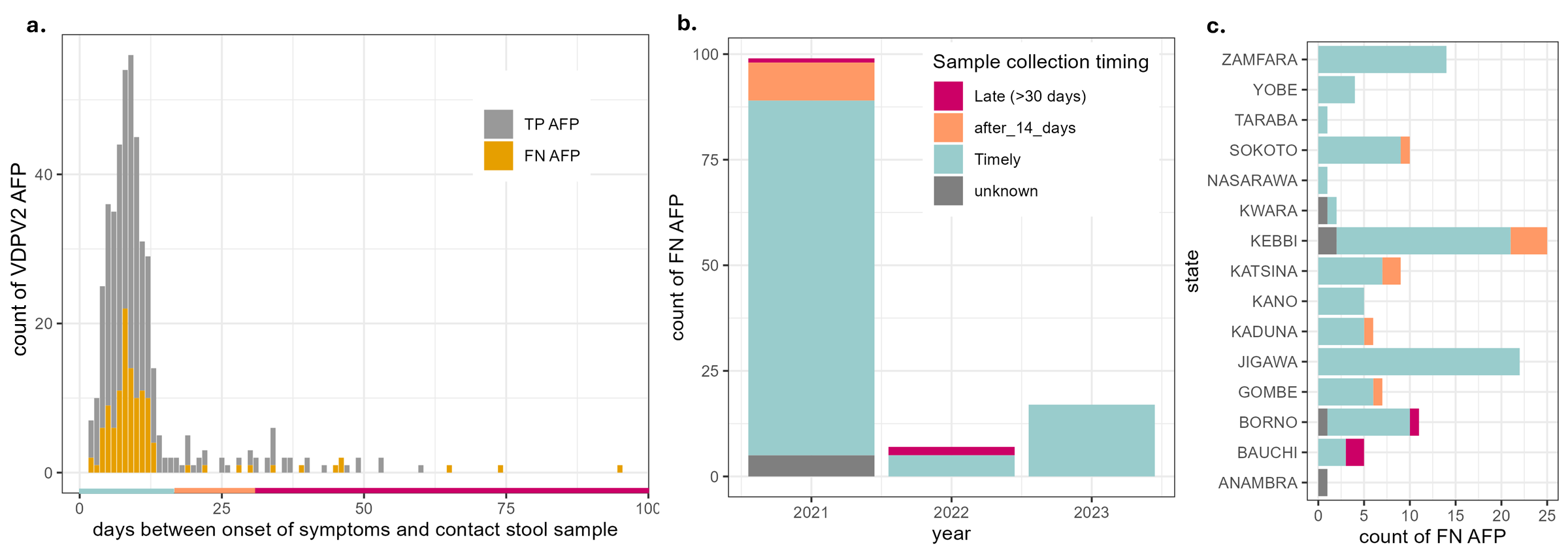

Supplement: S5 Fig — a. distribution of the delays to collect the VDPV2 AFP case ‘s contacts’ stool samples depending if they are of a True Positive (TP) AFP case in grey or a False Negative (FN) AFP case in gold; b. temporal distribution of the FN AFP case depending if their contacts’ stools have been collected late (more than 30 days after the onset) in pink, between 14 and 30 days after the onset in orange or timely in blue; spatial distribution of FN AFP cases depending if their contacts’ stools have been collected late (more than 30 days after the onset) in pink, between 14 and 30 days after the onset in orange or timely in blue. (TIF) [file pgph.0006371.s013.tif]

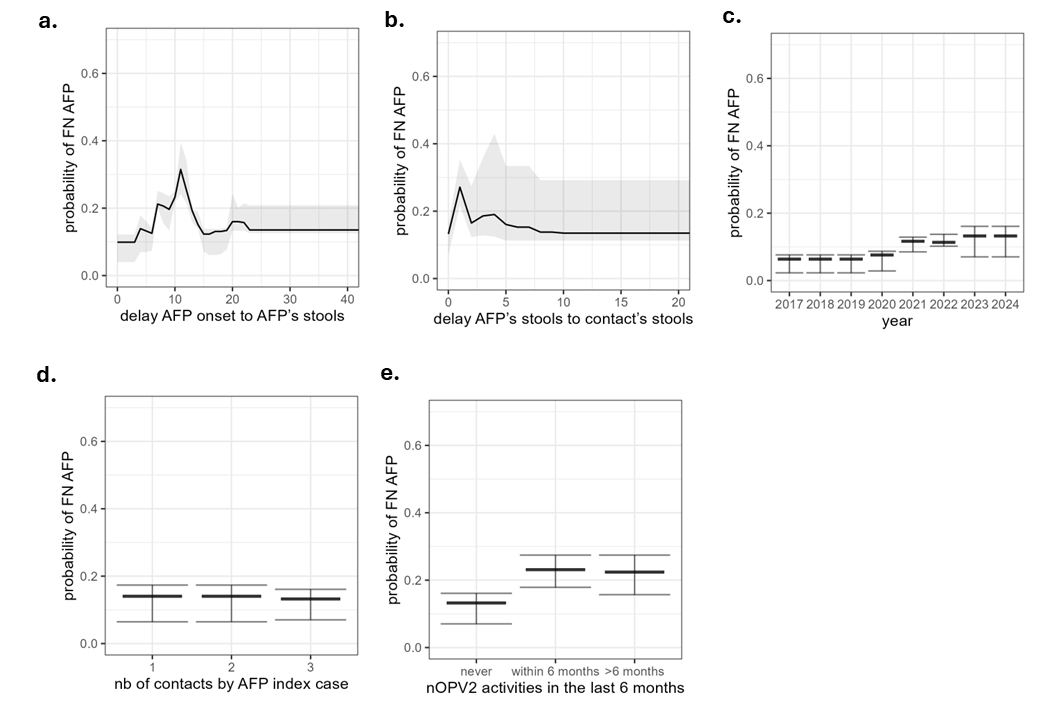

Supplement: S6 Fig — a. delay between AFPs onset and AFP case’s stool collection; b. delay between AFP case’s stool collection and contacts’ stool collection; c. year of paralysis onset; d. number of contact samples per AFP case; e. nOPV2 activities conducted in the state. (TIF) [file pgph.0006371.s014.tif]

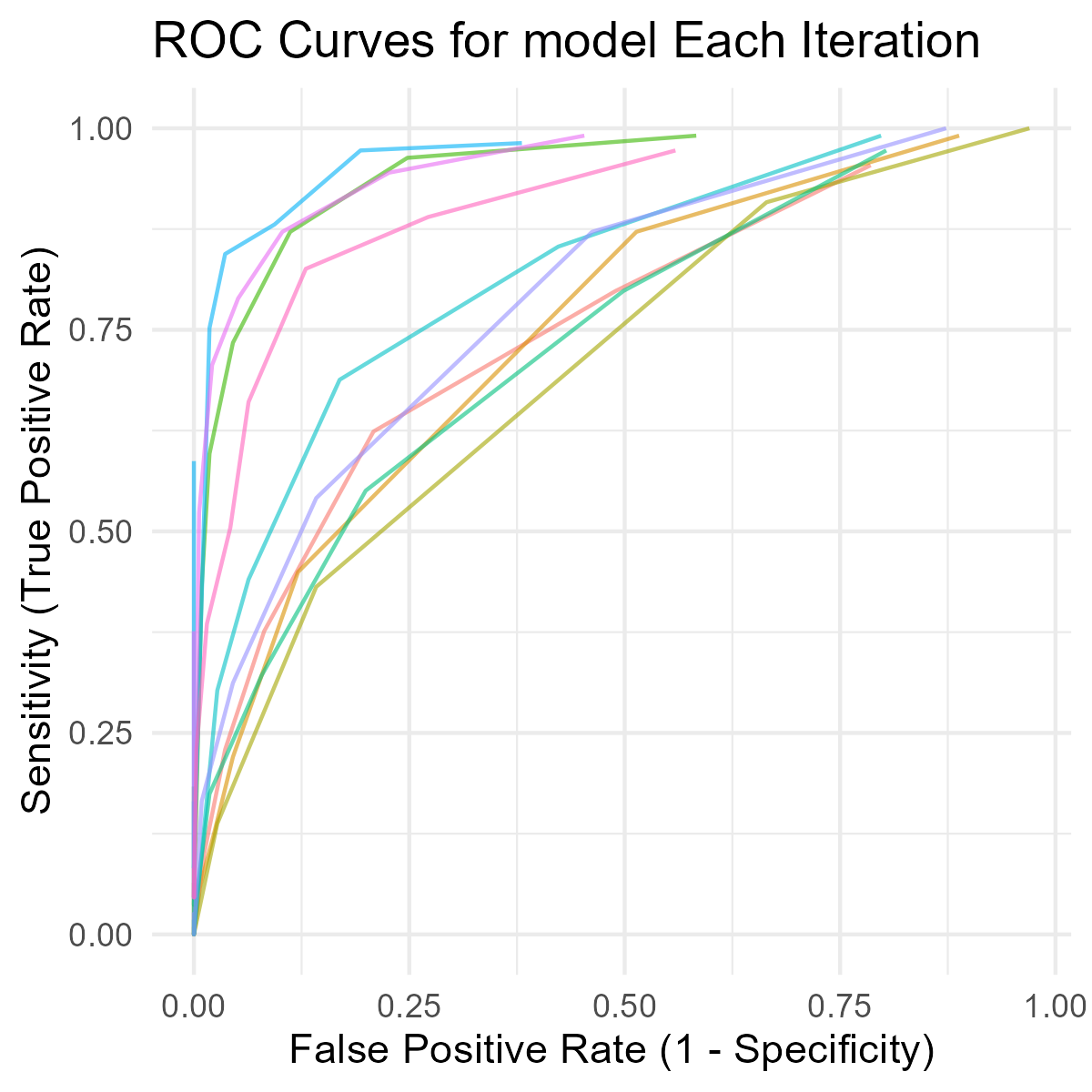

Supplement: S7 Fig — The 10 curves represented correspond to the 10 iterations of the model. (PNG) [file pgph.0006371.s015.png]
