# Supplementary material for: Quantifying the role of contact sampling for poliovirus detection in Nigeria
Source: PLOS Glob Public Health. 2026 May 13;6(5):e0006371. doi: 10.1371/journal.pgph.0006371 (PMC13170847; doi:10.1371/journal.pgph.0006371)
Supplement: Attachment — Submitted filename: Rev.docx [file pgph.0006371.s016.docx]

**Comments for Authors**

Dear authors,

I have no fundamental comments on your manuscript, which raises the important issue of increasing the sensitivity of AFP surveillance. Some comments of a technical nature and question for discussion:

**Introduction**

Lines 38-39. It is recommended to specify the types of eradicated polioviruses (types 2 and 3)

and to specify the type of wild poliovirus Type 1).

Line 40. Link 1 is incorrect. It is recommended to refer to the original documents that recorded these facts.

Lines 44, 47, Reference 2 - I believe that a more relevant reference to describe the situation would be not to the WHO information base, but to existing publications in scientific journals.

Line 66 - It is recommended to indicate the year when the last endemic wild poliovirus was detected in Nigeria.

Results

Table 1. It is recommended to add a TOTAL row in which to sum the numbers of each column.

Question:

Your study was conducted in Nigeria in the context of simultaneous circulation of several types of poliovirus type 2 (cVDPV2, viruses-derived from mOPV2, nOPV2 and mass immunization activities with using these vaccines. In such a situation on the one hand, , laboratory diagnostics becomes more complicated. On the other hand, there are known cases of poliomyelitis associated with the receipt of the vaccine (VAPP), including nOPV2. How can this affect the selection of healthy people (contacts) for the study? Is it possible to discuss this issue in this manuscript? For example, in the Discussion section.

**Comments for Editors**

Dear Editors,

The manuscript of *Dr Ledien J. and co-authors* is devoted to an important problem of Global Polio Eradication Initiative - increasing the sensitivity of polio surveillance. The authors convincingly demonstrate the importance of collection stool samples from healthy AFP contacts, which provides additional valuable information on the possible asymptomatic circulation of poliovirus. I have no fundamental remarks. Some technical comments and question are presented in the Comments for Authors. I recommend the manuscript for publication after minor technical corrections.
