# Supplementary material for: Quantifying the role of contact sampling for poliovirus detection in Nigeria
Source: PLOS Glob Public Health. 2026 May 13;6(5):e0006371. doi: 10.1371/journal.pgph.0006371 (PMC13170847; doi:10.1371/journal.pgph.0006371)
Supplement: Attachment — Submitted filename: Reply to reviewer comments .docx [file pgph.0006371.s018.docx]

Reply to reviewer comments

PGPH-D-25-01818

Title: Quantifying the role of contact sampling for poliovirus detection in Nigeria

Dear editor,

Thank you very much for providing us with the opportunity to revise our manuscript. We have addressed the reviewers’ comments as described below. In particular, we added additional explanations in the methods and have improved the clarity in the text. The paper is much improved as a result.

The line numbers provided in this document refer to the paper submitted with the track changes.

**Journal Requirements**:

Requirements 1

**Please provide additional details regarding participant consent. As you are reporting a retrospective study of medical records or archived samples, please ensure that you have discussed whether all data were fully anonymized before you accessed them and/or whether the IRB or ethics committee waived the requirement for informed consent. If patients provided informed written consent to have data from their medical records used in research, please include this information.**

Authors’ reply

Thank you for pointing out this was missing. We ‘ve added the information in part of the method describing the data.

We added line 153:

“All data were fully anonymised before access.

Requirements 2

**Please note that PLOS Global Public Health has specific guidelines on code sharing for submissions in which author-generated code underpins the findings in the manuscript. In these cases, all author-generated code must be made available without restrictions upon publication of the work. Please review our guidelines at https://journals.plos.org/globalpublichealth/s/materials-and-software-sharing# loc-sharing-code and ensure that your code is shared in a way that follows best practice and facilitates reproducibility and reuse.**

Authors’ reply

We agree with the editors’ comment and have added the link to our GitHub repository in the paper.

We added line 193:

“The R-code supporting the conclusions of this article is available in the repository in [34].”

With the reference [34] being:

“Ledien J. GitHub repository for quantifying the role of contact sampling for poliovirus detection in Nigeria n.d. https://github.com/mrc-ide/contact_sampling_polio_surveillance (accessed November 26, 2025).”

Reviewer #1:

Comment 1.1

**This article has a clear design and a useful aim for the GPEI. It has clear programmatic implications regarding conducting surveillance of contacts, and thus is a useful addition to the literature. I don't have any major substantive recommendations, and I think insight that contact sampling is useful in a particular window post-AFP onset is really useful. I have a few recommendations for clarity. The first is to frame a bit better who the paper is for (primarily people interested in polio eradication) and to clarify what literature exists on surveillance generally, and on contact surveillance specifically, and what this paper is adding.**

Authors’ reply

We thank the reviewer for their comment. We have now added two paragraphs in the introduction to describe the literature available on contact sampling in the context of poliovirus surveillance and have also clarified the added value of this study.

We added line 72:

“Most of the literature on AFP contact sampling includes polio surveillance reports or outbreak response activities where contact sampling is used to assess the extent of the outbreak [13–24]. Five studies assessed the benefits of contact sampling for the surveillance system [15,25–28], and only two studies, both in Mexico, followed household members to characterise the transmission between close contacts and to other community members [10,29]. Results suggest that when a poliovirus is detected, either with wild-type, attenuated vaccine or vaccine-derived poliovirus, there is around a 20% chance that another household member would shed the virus as well. Only two studies reported poliomyelitis cases confirmed solely through identification of poliovirus in their contacts’ stools (i.e. false negative cases) [9,25]. In India, contact sampling revealed poliovirus-positive contacts in 2.2% to 2.6% of the AFP cases [9]. During the wild poliovirus outbreak in Somalia (2013), 38% of their AFP cases were false negative and were confirmed through contact sampling [25].

Contact sampling is an important surveillance tool for the GPEI, as it can be deployed quickly whenever and wherever outbreaks happen. It complements environmental surveillance, which relies on longitudinal wastewater testing, but only is applicable to large portions of populations who are connected to a convergent sewage network. Extended and systematic contact sampling has rarely been done due to the increased cost and laboratory workload, data on real-world polio contacts are scarce, and studies on the best strategies to implement it are non-existent.”

Also, on line 102, we wrote:

“The extensive implementation of contact sampling in Nigeria offers a unique opportunity to assess the value of this surveillance method to GPEI and how it can be optimised to enhance surveillance sensitivity. Here, we aim to provide an insight into the importance of contact sampling for the detection of poliovirus and assess the optimal conditions for implementation. “

Comment 1.2

**The second is to review the language throughout for clarity. I have some suggestions for the first few pages pasted below; I suggest a careful read of the paper for clarity and flow. Some of the paper authors have other work that is very clear and easy to read, so I suggest the full author list be brought to bear to assist here.**

Authors’ reply

We thank the reviewer for pointing out those flows. The manuscript has been reviewed thoroughly to improve the flow and clarity.

Comment 1.3

**Some examples of places from the first few pages where further editing could be useful (this type of thing is needed throughout the paper): There were a few places in the abstract where I found the language convoluted and confusing. For example I would recommend rewording the second sentence as “****Collecting stool samples from healthy close contacts of AFP cases, preferably contacts aged under 5 years, is a potential method for enhancing surveillance.”**

Authors’ reply

We thank the reviewer for their suggestion. The sentence has been changed accordingly.

In the abstract, line 16:

“Surveillance enhancement is possible when collecting stool samples from healthy contacts (preferably aged under 5 years old) who must have been in close contact with the AFP case. ”

Was replaced by:

“Collecting stool samples from healthy close contacts of AFP cases, preferably contacts aged under 5 years, is a potential method for enhancing surveillance.”

Comment 1.4

**I would also add in the abstract that this study aimed to determine whether this contact surveillance was useful!**

Authors’ reply

We thank the reviewer for their suggestion.

We added line 24:

“Here we analysed data from 71,002 AFP cases and 51,100 associated contacts sampled in Nigeria between January 2017 and November 2023 to quantify the added value of contact sampling and assess how it can be optimised. ”

Comment 1.5

**First paragraph of the introduction: need to mention that the one wild serotype that was eradicated is Type 2, which was the rationale for taking it out of OPV in 2016.**

Authors’ reply

We thanks the reviewer for their suggestion, and we’ve modified the paragraph accordingly.

In the introduction, line 40:

“The Global Polio Eradication Initiative (GPEI) has eradicated two out of three serotypes of wild poliovirus and has reduced circulation of the last wild serotype to Pakistan and Afghanistan [1]. This has been achieved largely through the use of the live-attenuated oral polio vaccine (OPV). However, the vaccine virus can revert to a virulent poliovirus and seed new outbreaks of circulating vaccine-derived poliovirus (cVDPV) [1]. Most of those outbreaks have been caused by serotype 2 (cVDPV2), and 44 countries have experienced outbreaks following the global withdrawal of serotype 2 from OPV in 2016, with 32 located on the African continent [2]. Although a type-2 novel OPV (nOPV2) has been developed to be more genetically stable and has been used in outbreak response since 2021, outbreaks have still emerged from its use, albeit at a lower rate [2]. ”

Was replaced by:

“The Global Polio Eradication Initiative (GPEI) has reduced circulation of the last remaining wild poliovirus serotype (serotype 1) to Pakistan and Afghanistan [1]. Eradication of wild poliovirus serotypes 2 and 3 were certified in 2015 and 2019 respectively [2]. These milestones have been achieved largely through the use of the live-attenuated oral polio vaccine (OPV). However, the vaccine virus can revert to a virulent poliovirus and seed new outbreaks of circulating vaccine-derived poliovirus (cVDPV) [3]. Most of those outbreaks have been caused by serotype 2 (cVDPV2), which was withdrawn from OPV in 2016 to complete its eradication [4]. Although a type-2 novel OPV (nOPV2) has been developed to be more genetically stable and has been used in outbreak response since 2021, outbreaks have still emerged from its use, albeit at a lower rate [5,6].”

Comment 1.6

**Line 58: Please add a “the” at the beginning of this sentence Etc, throughout the paper.**

Authors’ reply

We thank the reviewer for pointing out this mistake. The manuscript has been revised throughout to improve the grammar.

Comment 1.7

**Overall this paper is a useful contribution to the literature and with some attention to presentation can helpfully inform policy.**

Authors’ reply

We thank the reviewer for the time spent to help us improve this manuscript.

Reviewer #2:

Comment 2.1

**Dear authors, I have no fundamental comments on your manuscript, which raises the important issue of increasing the sensitivity of AFP surveillance. Some comments of a technical nature and question for discussion.**

**Lines 38-39. It is recommended to specify the types of eradicated polioviruses (types 2 and 3) and to specify the type of wild poliovirus Type 1).**

Authors’ reply

We agree with the reviewer’s comment and have modified the paragraph accordingly.

In the introduction, line 40:

“The Global Polio Eradication Initiative (GPEI) has eradicated two out of three serotypes of wild poliovirus and has reduced circulation of the last wild serotype to Pakistan and Afghanistan [1]. This has been achieved largely through the use of the live-attenuated oral polio vaccine (OPV). However, the vaccine virus can revert to a virulent poliovirus and seed new outbreaks of circulating vaccine-derived poliovirus (cVDPV) [1]. Most of those outbreaks have been caused by serotype 2 (cVDPV2), and 44 countries have experienced outbreaks following the global withdrawal of serotype 2 from OPV in 2016, with 32 located on the African continent [2]. Although a type-2 novel OPV (nOPV2) has been developed to be more genetically stable and has been used in outbreak response since 2021, outbreaks have still emerged from its use, albeit at a lower rate [2]. ”

Was replaced by:

“The Global Polio Eradication Initiative (GPEI) has reduced circulation of the last remaining wild poliovirus serotype (serotype 1) to Pakistan and Afghanistan [1]. Eradication of wild poliovirus serotypes 2 and 3 were certified in 2015 and 2019 respectively [2]. These milestones have been achieved largely through the use of the live-attenuated oral polio vaccine (OPV). However, the vaccine virus can revert to a virulent poliovirus and seed new outbreaks of circulating vaccine-derived poliovirus (cVDPV) [3]. Most of those outbreaks have been caused by serotype 2 (cVDPV2), which was withdrawn from OPV in 2016 to complete its eradication [4]. Although a type-2 novel OPV (nOPV2) has been developed to be more genetically stable and has been used in outbreak response since 2021, outbreaks have still emerged from its use, albeit at a lower rate [5,6].

Comment 2.2

**Line 40. Link 1 is incorrect. It is recommended to refer to the original documents that recorded these facts.**

Authors’ reply

We thank the reviewer for their suggestion.

The first reference:

“Cooper LV, Erbeto TB, Danzomo AA, Abdullahi HW, Boateng K, Adamu US, et al. Effectiveness of poliovirus vaccines against circulating vaccine-derived type 2 poliomyelitis in Nigeria between 2017 and 2022: a case-control study. Lancet Infect Dis 2024;24:427–36. https://doi.org/10.1016/S1473-3099(23)00688-6.”

Has been replaced by:

“Wassilak SGF, Mohamed A, Bigouette JP, Wassilak SGF, Mohamed A, Bigouette JP. Impediments to Progress Toward Polio Eradication During 2014–2024: Effectively Addressing the Current Challenges. Vaccines 2025;13. <https://doi.org/10.3390/vaccines13101060>.”

Comment 2.3

**Lines 44, 47, Reference 2 - I believe that a more relevant reference to describe the situation would be not to the WHO information base, but to existing publications in scientific journals.**

Authors’ reply

We agree with the reviewer and replaced the second reference:

“About POLIS n.d. https://extranet.who.int/polis/About (accessed June 27, 2024).”

With:

“[3]	Cooper LV, Erbeto TB, Danzomo AA, Abdullahi HW, Boateng K, Adamu US, et al. Effectiveness of poliovirus vaccines against circulating vaccine-derived type 2 poliomyelitis in Nigeria between 2017 and 2022: a case-control study. Lancet Infect Dis 2024;24:427–36. https://doi.org/10.1016/S1473-3099(23)00688-6.

[4]	Macklin GR, Goel AK, Mach O, Tallis G, Ahmed JA, O’Reilly KM, et al. Epidemiology of type 2 vaccine-derived poliovirus outbreaks between 2016 and 2020. Vaccine 2023;41 Suppl 1:A19–24. https://doi.org/10.1016/j.vaccine.2022.08.008.

[5]	Peak CM, Lyons H, Voorman A, Gray EJ, Cooper LV, Blake IM, et al. Monitoring the Risk of Type-2 Circulating Vaccine-Derived Poliovirus Emergence During Roll-Out of Type-2 Novel Oral Polio Vaccine. Vaccines 2024;12:1308. https://doi.org/10.3390/vaccines12121308.

[6]	Bandyopadhyay AS, Cooper LV, Zipursky S. One billion doses and WHO prequalification of nOPV2: Implications for the global polio situation and beyond. PLOS Glob Public Health 2024;4:e0002920. https://doi.org/10.1371/journal.pgph.0002920.

Comment 2.4

**Line 66 - It is recommended to indicate the year when the last endemic wild poliovirus was detected in Nigeria.**

Authors’ reply

We thank the reviewer for their suggestion. The Nigerian situation is described on line 94:

“Nigeria was the last country to stop endemic wild poliovirus transmission in Africa in 2016, and AFP contact sampling was expanded in this final year following the detection of type-1 wild poliovirus after a two-year gap in observed circulation. Nigeria has also reported the largest number of cVDPV2 cases from the region and was the first country to introduce nOPV2 under EUL in March 2021 [3,30]. ”

Comment 2.5

**Table 1. It is recommended to add a TOTAL row in which to sum the numbers of each column.**

Authors’ reply

We thank the reviewer for their suggestions.

Line 223, table 1:

*Table 1: Distribution of the number of AFP cases and their contacts in Nigeria with stool samples collected between the 1st January 2017 and the 30th November 2023.*

| *Year of stool collection* | *Number of AFP cases* | *Number of AFP with contacts* | *Number of contacts collected* | *Number of VDPV2 positive AFP1* | *Number of VDPV2 positive contacts* |
| --- | --- | --- | --- | --- | --- |
| *2017* | *16,762* | *1,966* | *5,555* | *0* | *0* |
| *2018* | *9,767* | *1,643* | *4,655* | *34* | *5* |
| *2019* | *7,645* | *1,488* | *4,259* | *25* | *5* |
| *2020* | *6,447* | *1,141* | *3,228* | *8* | *8* |
| *2021* | *7,955* | *3,908* | *11,350* | *425* | *312* |
| *2022* | *10,491* | *4,026* | *11,303* | *46* | *31* |
| *2023* | *11,333* | *3,745* | *10,750* | *62* | *41* |

*1 data extracted from POLIS on the 8th of January 2024*

Was replaced with:

“Table 1: Distribution of the number of AFP cases and their contacts in Nigeria with stool samples collected between the 1^st^ January 2017 and the 30^th^ November 2023.

| **Year of stool collection** | **Number of AFP cases^1^** | **Number of AFP with contacts** | **Number of contacts collected** | **Number of VDPV2 positive AFP^1,2^** | **Number of VDPV2 positive AFP^1,2^ having contacts** | **Number of VDPV2 positive contacts** |
| --- | --- | --- | --- | --- | --- | --- |
| 2017 | 16,762 | 1,966 | 5,555 | 0 | 0 | 0 |
| 2018 | 9,767 | 1,643 | 4,655 | 34 | 15 | 5 |
| 2019 | 7,645 | 1,488 | 4,259 | 25 | 10 | 5 |
| 2020 | 6,447 | 1,141 | 3,228 | 8 | 3 | 8 |
| 2021 | 7,955 | 3,908 | 11,350 | 425 | 339 | 312 |
| 2022 | 10,491 | 4,026 | 11,303 | 46 | 37 | 31 |
| 2023 | 11,333 | 3,745 | 10,750 | 62 | 54 | 41 |
| **TOTAL** | **70,400** | **17,917** | **51,100** | **600** | **458** | **402** |

^1^ data extracted from POLIS on the 8^th^ of January 2024

^2^ including true positive VDPV2 AFP (total n= 477) and false negative VDPV2 AFP (total n=123)”

Comment 2.6

**Your study was conducted in Nigeria in the context of simultaneous circulation of several types of poliovirus type 2 (cVDPV2, viruses-derived from mOPV2, nOPV2 and mass immunization activities with using these vaccines. In such a situation on the one hand, , laboratory diagnostics becomes more complicated. On the other hand, there are known cases of poliomyelitis associated with the receipt of the vaccine (VAPP), including nOPV2. How can this affect the selection of healthy people (contacts) for the study? Is it possible to discuss this issue in this manuscript? For example, in the Discussion section.**

Authors’ reply

We agree with the reviewer that the multiple circulation of serotypes and the various immunisation levels complicate the picture. Regarding the laboratory diagnostics, standardised methods rely on cell culture followed by sequencing. If adding cost and complexity, the sequencing step easily distinguishes cVDPV from Sabin-derived serotypes. It also distinguishes the cVDPV emergence group, the vaccine it is derived from, the dates of emergence, and the potential extent of the outbreak. The same laboratory methods are used throughout the whole analysis period and systematically across AFP and contact stool samples.

AFP contacts are selected before the cause of AFP is determined (i.e. there is not differential contact sampling for WPV1, cVDPV2, VAPP or non-polio AFP cases). Vaccination status of the case or contacts is not considered. The only criteria is that contacts are ideally children of the same or younger age belonging to the same household or friend group. The majority of AFP cases are non-polio AFP cases and vaccine virus is detected from their stool as a coincidence of vaccination prior to developing AFP from a different cause.

We have made it clearer in the text that the selection of contacts is independent of the cause of AFP. On line 378, we added:

“The selection and sampling of AFP contacts is independent to the aetiology of AFP (i.e. Wild poliovirus, cVDPV2, Vaccine-associated paralytic poliomyelitis (VAPP), or non-polio AFP) and vaccination status of cases and contacts. Poliovirus transmission between contacts and detection of the virus in stool is associated with age, immunisation status, transmission intensity and timing of stool collection [9,29,36].”

Reviewer #3:

Comment 3.1

**This is a good analysis of the unique AFP and contact sampling available within the GPEI partners. The problem is presented well, ie. We know that early detection of outbreaks are important but AFP alone isn’t so good for a variety of reasons. The concept of contact sampling is well presented. The methods are good, but I would encourage the authors to explain what and why they did in more detail. I don’t get a sense of how important contact sampling is for early detection, and some simple analysis could perhaps help with this. This research is an important question for those involved in polio eradication, whom are numerous, and has policy implications, especially toward progress made in eradication.**

Authors’ reply

We thank the reviewer for their comments, and, as suggested, we have added more details to the methods section. See the following detailed responses as well.

Comment 3.2

**In reference to false negative and true positive AFP, best to have definitions as a full sentence, rather than parenthesis.**

Authors’ reply

We agree with the reviewer and modified the sentence accordingly.

On line 135:

“An AFP case is classified as a VDPV2 case if the virus is detected in their stool or if their stool is negative but one of their contacts has VDPV2-positive stools (we term the latter as a false negative AFP, while the AFP with positive stools are termed true positive AFP).”

Was replaced by:

“An AFP case is classified as a VDPV2 case if the virus is detected in their stool or if their stool is negative but one of their contacts has VDPV2-positive stools. We term the latter as a false negative AFP, while the AFP cases with positive stools are termed a true positive AFP.”

Comment 3.3

**“Before 2021, only AFP cases with inadequate stools could be classified as false negatives”. More detail needed, I think referring to the process of identifying “compatible” cases?**

Authors’ reply

Compatible cases are those whose clinical symptoms are similar to those of poliomyelitis (according to the National Polio Expert Committee) but whose stool samples were inadequate or were not collected. If associated contact stool samples were collected and cVDPV / WPV1 was isolated the respective AFP would be then be classified as poliomyelitis. What has changed since 2021 is that if AFP stool samples are adequately collected but cVDPV is not isolated but it is isolated from associated contact stools then the AFP is categorised as a cVDPV poliomyelitis case. Compatible classification occurs after contact stool samples are tested so we prefer to not discuss compatible case classification (eg see [here](chrome-extension://efaidnbmnnnibpcajpcglclefindmkaj/https:/polioeradication.org/wp-content/uploads/2023/03/Global-AFP-guidelines-pre-publiucation-version-2023.pdf)) here as it is not relevant to our analysis.

Comment 3.4

**Poliovirus serotype-specific concordance between AFP cases and their contacts: Was cVDPV2 emergence group not available? Please confirm as this would help understand concordance better.**

Authors’ reply

When we studied the concordance, we didn’t have all the data to distinguish between Sabin and cVDPV2 for all contacts. We restricted the analyses to the poliovirus serotype to include as much contact data as possible. The S2 Fig describe that the sequencing results were missing for 371 contacts out of the 755 having positive culture results for PV2. Regarding the emergence groups, analysing them was not very relevant, as there were only three emergence groups circulating during the study period. We have added the S2 Table to describe the emergence groups in Nigeria between 2015 and 2023. We also added more detail on emergence groups in the main text:

In the results, line 241, we added:

“In 2019, the NIE-SOS-7 cVDPV2 emergence was detected in Nigeria and among the first 10 detections, three were made from contact [35]. In 2020, when NIE-SOS-8 emerged, contacts accounted for 7 of the first 10 detections (S2 Table).”

With, in the S2 Table:

S2 Table: Surveillance system provenance of the first 10 detections for each emergence group that has affected Nigeria between 2015 and 2023. The surveillance type refers to the surveillance system through which the virus was detected

| *Emergence group* | *year of first detection* | *Total number of detections* | *surveillance type for the first 10 detections* | | | |
| --- | --- | --- | --- | --- | --- | --- |
|  |  |  | *AFP* | *contact* | *ES* | *other* |
| *NIE-SOS-2* | *2016* | *2* | *1* | *1* | *0* | *0* |
| *NIE-JIS-1* | *2018* | *265* | *3* | *0* | *7* | *0* |
| *NIE-SOS-3* | *2018* | *26* | *0* | *0* | *10* | *0* |
| *NIE-SOS-7* | *2019* | *58* | *3* | *3* | *4* | *0* |
| *NIE-KGS-1* | *2019* | *8* | *3* | *0* | *4* | *1* |
| *NIE-SOS-6* | *2019* | *5* | *0* | *0* | *5* | *0* |
| *NIE-KGS-2* | *2019* | *5* | *2* | *0* | *0* | *3* |
| *NIE-SOS-5* | *2019* | *2* | *1* | *0* | *0* | *1* |
| *NIE-SOS-4* | *2019* | *4* | *0* | *0* | *4* | *0* |
| *NIE-ZAS-1* | *2020* | *1112* | *6* | *0* | *4* | *0* |
| *NIE-SOS-8* | *2020* | *11* | *2* | *7* | *1* | *0* |
| *NIE-KBS-1* | *2021* | *3* | *3* | *0* | *0* | *0* |

Also, in the Discussion, line 408, we added:

“Finally, studying concordance between AFP and contact at the cVDPV2 emergence group level could have brought some insight into the transmission dynamics. However, our analyses were limited to the Nigerian context, where there is little overlap in geography or time across emergence groups.”

Comment 3.5

**It’s not clear to me from the text why a BRT approach was taken. There are other much more intuitive and simpler modelling approaches, ie. GLMs, GAMs that should really be considered. At the minimum, a justification of why this methodological approach was taken. Where other trialled and not informative? I would be much more comfortable seeing alongside an analysis using a simpler model framework. Otherwise the approach can come across as quite a ‘blackbox’. Some more details is provided in the SM, some should be in the main text. The figures need more explanation.**

Authors’ reply

We agree with the reviewer that the choice in the model was not explicit. The motivation to choose this model relied on the nature of the data and the question to answer. The predictors used in those models were collinear, and their relationship with the response variable was not linear. Furthermore, the shape of the relationship was of interest to us, i.e., what is the time window of importance? Considering we had no prior knowledge of this relationship's shape or the collection time window that would matter, the machine learning approach was the more straightforward method. Then, among the machine learning models, we chose the BRT because we were more interested in explaining as much variance as possible (getting the maximum amount of information from the data) than making predictions. Compared to other models like Random Forest, BRT has a process that tries to maximise the variance that can be explained ( at each iteration, the variable and threshold selected is the one that best improves the model). In Random Forest, this process is made random to improve robustness.

To clarify our approach in the paper, we’ve added some of the details previously in the supplement into the main text.

In the method section, line 164:

“We identified factors associated with the probability of finding i) a VDPV2 contact, or ii) a false negative AFP through fitting ensemble Boosted Regression Trees (BRT) models. Details on the model and a flow diagram are available in S1 Text and S1 Fig. In brief, the dataset was divided into a fitting and cross-validation (CV) set; a BRT model was built using the fitting set, and the performance was assessed based on spatial resampling and the CV set. This process was iterated to build an ensemble model. ”

Was replaced by:

“We identified factors associated with the probability of finding i) a VDPV2 contact, or ii) a false negative AFP through fitting ensemble Boosted Regression Trees (BRT) models. This method was selected due to its flexibility in handling collinearity among the factors and because it does not require linear assumptions. Also, the shape of the relationship between the response variable and the factors was our main interest. The ensemble models were developed through a series of BRT iterations. In each iteration, the data were resampled and divided into two subsets: the fitting set, which was used to tune hyperparameters, fit, and test the model, and the cross-validation (CV) set, which assessed the model's performance using data not involved in the fitting process. For each BRT model built, the contribution of each factor to the model (called factor’s importance) was assessed. Random and spatial resampling were realised to test the model's performance and check for spatial autocorrelation. Predictions were generated for the CV set to evaluate the model's accuracy. Additionally, the marginal effects of the predictors on the response variable were calculated to visualise how the probability of finding a positive contact or false negative varies across the range of values that each predictor could take. More details on the models and a flow diagram are available in S1 Text and S1 Fig.”

Comment 3.6

**Fig 2a reports “relative importance” as an outcome. It is not clear what this is and requires explanation. For example this paper from ecology describes some approaches to interpretation of BRT importance, and note they recommend importance is scaled up to 100% https://besjournals.onlinelibrary.wiley.com/doi/10.1111/j.1365-2656.2008.01390.x - and actually this is cited in the SM...! So please scale as described.**

Authors’ reply

We agree with the reviewer that this outcome needs more explanation and that scaling up the relative importance helps to understand it better. We modified Figs 2and 3 accordingly. Also, we changed the label of the axis to “relative contribution (%)” to improve clarity and modified the text to add the definition of the relative importance:

In the results, line 265, we added:

Fig. 2 describes the results from the BRT model assessing the probability of VDPV2 contact when the AFP is confirmed as VDPV2. Fig. 2.a shows the factors’ relative contribution, which measures how often the model uses each factor and how much variance it explains, giving a sense of the factor's weight in the model. Here, it is represented as a proportion of the total contribution (i.e., the sum of all factors’ contributions equals 100%). Fig. 2.b, Fig. 2.c, and Fig. 2.d show the marginal effects of selected factors, indicating how the response variable changes within the range of values that the factor can take, while all other factors are held constant.

Line 281, the legend of the fig 2:

“Fig 2: Relative importance and marginal effects of the factors included in the Boosted Regression Tree ensemble model assessing the probability of a VDPV2 positive contact when the index AFP case is VDPV2 positive themselves. a. relative importance of all of the included factors (nb = number, TP = true positive); b. marginal effects of the delay in days between AFP onset and contacts’ stools sampling; c. marginal effects of the age of the AFP case; d. number of true positive AFP cases in the last month. The marginal effects of the other factors are presented in S3 Fig.“

Was replaced by:

“Fig 2: Relative contribution and effects of the factors included in the Boosted Regression Tree ensemble model assessing the probability of a VDPV2 positive contact when the index AFP case is VDPV2 positive themselves. a. relative contribution of the factors scaled to 100% (nb = number, TP = true positive); b. marginal effects of the delay in days between AFP onset and contacts’ stools sampling; c. marginal effects of the age of the AFP case; d. number of true positive AFP cases in the last month. The marginal effects of the other factors are presented in S3 Fig.”

Equally, line 319, the legend of Fig 3:

Fig 3: Relative importance and marginal effects of the factors included in the Boosted Regression Tree ensemble model of the probability of a false negative AFP case in Nigeria. a. relative importance of all of the factors included (nb = number, TP = true positive); b. marginal effects of the age of the AFP; c. marginal effects of the number of true positive AFP cases in the last month; d. marginal effects of the delay in days between AFP onset and AFP stools sampling for several values of the delay between the collection of stools from the AFP and those of the contacts. The marginal effects of the other factors are presented in S5 Fig.”

Was replaced by:

“Fig 3: Relative importance contribution and marginal effects of the factors included in the Boosted Regression Tree ensemble model of the probability of a false negative AFP case in Nigeria. a. relative importance contribution of all of the factors included scaled to 100% (nb = number, TP = true positive); b. marginal effects of the age of the AFP; c. marginal effects of the number of true positive AFP cases in the last month; d. marginal effects of the delay in days between AFP onset and AFP stools sampling for several values of the delay between the collection of stools from the AFP and those of the contacts. The marginal effects of the other factors are presented in S5 Fig. ”

Comment 3.7

**Fig 2b and c. Not clear what ‘marginal effects’ are, do you mean partial dependency plots? Again, how to interpret these needs to be explained better. I would also recommend adding ticks on the x-axes to indicate where the data lie.**

Authors’ reply

We appreciate the reviewer’s feedback regarding Figures 2b and 2c, noting that they are partial dependence plots and that the concept of marginal effects requires further clarification. In machine learning, marginal effects refer to how the response variable changes within the range of values that a predictor can take when all other predictors are held constant. Partial dependence plots illustrate these marginal effects of the predictors on the response variable, so both terminologies are appropriate. We have added a definition of marginal effects to the figure legend and resized the ticks, as suggested.

In the results, line 265, we added:

Fig. 2 describes the results from the BRT model assessing the probability of VDPV2 contact when the AFP is confirmed as VDPV2. Fig. 2.a shows the factors’ relative contribution, which measures how often the model uses each factor and how much variance it explains, giving a sense of the factor's weight in the model. Here, it is represented as a proportion of the total contribution (i.e., the sum of all factors’ contributions equals 100%). Fig. 2.b, Fig. 2.c, and Fig. 2.d show the marginal effects of selected factors, indicating how the response variable changes within the range of values that the factor can take, while all other factors are held constant.

Comment 3.8

**For the analysis explored factors associated with a false negative case, it is not clear what data are used to fit this model. I understand that there are 123-9=114 false negatives, is this the only data used? The authors need to be more clear on the total data, and the comparator / what is trying to be classified.**

Authors’ reply

Apologies this wasn’t clear. We have now modified the text to be explicit about the data used in this analysis:

We added line 306:

“To model the probability of a VDPV2 AFP being a false negative AFP, all the VDPV2 AFP with 1 to 3 contacts were included in this analysis (N = 440). Among them, 25% (n=109/440) were false negative AFP, and 75% (n=331/440) were true positive.”

Comment 3.9

**I would recommend summary stat of effort required to find an additional case of i) true positive AFP, ii) and false negative AFP by using contact sampling. In the 2021+ data, how many AFP cases were identified for every true positive AFP, ie. Total AFP / true positive AFP = 29779 / 533 = 55 cases for every polio case found**

Authors’ reply

We thank the reviewer for their suggestion.

We added line 236:

“Since the start of enhanced surveillance in 2021, a total of 29,779 AFP cases have been identified, among which 533 were confirmed as VDPV2 polio cases. This indicates that for every polio-positive case, 55 AFP cases are investigated on average.”

We respond to the FN question in the next response.

Comment 3.10

**And for the false negative, total contacts of AFP / false negative found = 33403 / 123 = 272 contacts for every extra polio case found. I appreciate this is crude, but simply calculated illustrates the additional effort needed to identify additional cases using contact sampling. As a additional surveillance tool, it is very resource intensive, and the prior uses have been for specific outbreaks with known issues in surveillance quality (this should be highlighted).**

Authors’ reply

We thank the reviewer for their suggestion.

We added line 238:

“Additionally, 33,403 contacts were tested, resulting in the identification of 123 false negative VDPV2 AFP cases, meaning that an average of 271 contacts were tested for each missed VDPV2 AFP case.”

Comment 3.11

**I don’t get a sense of the role that contact sampling may have played in the critical cases, as in not all cases are equally important when considering rapid detection of cVDPV2 emergence (as posed in the introduction). For example, of the cVDPV2 emergences that were detected in Nigeria between 2017 - 2023, for how many were contact samples included in the first (say) 10 cases? This would help better understand the role in early detection. From the analysis presented I don’t get a sense of whether the contact samples are cases within a spatially defined epidemic, or providing information early on in the epidemic. If it is the former, it is still important in terms of cases count and understanding local transmission, but perhaps less critical for early detection.**

Authors’ reply

We agree with the reviewer that this is a very important point.

We’ve added the S2 Table, which describes the emergence groups detected in Nigeria between 2015 and 2023 and the type of surveillance (AFP, ES, contact or other) from which the first 10 detections were identified. Only 3 emergence groups were detected between 2020 and 2022 (NIE-ZAS-1, NIE-SOS-8, and NIE-KBS-1) , and only 1 from 2021 (NIE-KBS-1), when the surveillance enhancement had started, which had only 3 AFP detected in total. The NIE-SOS-8 had a good contribution from the contact surveillance. This is related to the NIE-SOS-7, which started in 2019 but then circulated silently for 6 months, triggering contact sampling in the area when new cases were detected in 2020.

On the other hand, ES is very well developed in Nigeria with 199 sites across the country. This is not the case in other African countries. Comparing the first 10 detections in other contexts would help to understand the best strategy to combine ES and contact sampling to improve early detection of outbreaks.

*S2 Table: Surveillance system provenance of the first 10 detections for each emergence group that has affected Nigeria between 2015 and 2023. The surveillance type refers to the surveillance system through which the virus was detected*

| *Emergence group* | *year of first detection* | *Total number of detections* | *surveillance type for the first 10 detections* | | | |
| --- | --- | --- | --- | --- | --- | --- |
|  |  |  | *AFP* | *contact* | *ES* | *other* |
| *NIE-SOS-2* | *2016* | *2* | *1* | *1* | *0* | *0* |
| *NIE-JIS-1* | *2018* | *265* | *3* | *0* | *7* | *0* |
| *NIE-SOS-3* | *2018* | *26* | *0* | *0* | *10* | *0* |
| *NIE-SOS-7* | *2019* | *58* | *3* | *3* | *4* | *0* |
| *NIE-KGS-1* | *2019* | *8* | *3* | *0* | *4* | *1* |
| *NIE-SOS-6* | *2019* | *5* | *0* | *0* | *5* | *0* |
| *NIE-KGS-2* | *2019* | *5* | *2* | *0* | *0* | *3* |
| *NIE-SOS-5* | *2019* | *2* | *1* | *0* | *0* | *1* |
| *NIE-SOS-4* | *2019* | *4* | *0* | *0* | *4* | *0* |
| *NIE-ZAS-1* | *2020* | *1112* | *6* | *0* | *4* | *0* |
| *NIE-SOS-8* | *2020* | *11* | *2* | *7* | *1* | *0* |
| *NIE-KBS-1* | *2021* | *3* | *3* | *0* | *0* | *0* |

Comment 3.12

**I’m less convinced that contact sampling is ‘critical’, or has a ‘clear value’, as phrased in the Discussion. Indeed surveillance is critical but doing it in a meaningful and pragmatic way is important. Surveillance is only critical because of the actions taken as a result of what was detected. Going back to my “more general” point, did the contact sampling result in declaring an outbreak earlier than relying on AFP alone?**

Authors’ reply

We agree with the reviewer that assessing the clear contribution of contacts in the declaration of outbreaks would be valuable. However, contact sampling is typically conducted after an outbreak has been declared to better understand its extent. The main result of our paper highlighted the added value of contact sampling in identifying additional acute flaccid paralysis (AFP) cases that were confirmed as polio cases. This raises questions about the sensitivity of the surveillance system. Since AFP cases account for less than 1% of polio infections, can we afford to miss any of them? Also, when contact sampling is employed, defining the best time window to do it is a valuable information.

Line 433:

“In the context of reaching eradication and the wide use of vaccines that reduce poliovirus shedding duration, increasing surveillance sensitivity through contact sampling to detect virus circulation fast is critical.”

Was replaced by:

“Contact sampling and environmental surveillance are complementary surveillance strategies to increase surveillance sensitivity. New molecular tools could help reduce diagnostic processing time and increase testing capacity, allowing for an increased scale of contact sampling [38]. Between 2015 and 2023, AFP contact sampling only contributed to the first 10 detections of VDPV2 outbreaks in three out of twelve outbreaks, although most outbreaks emerged outside the period of enhanced AFP contact sampling. Further work can investigate how this surveillance method contributes to defining the scope of response and understanding of spatial spread. However, eight out of the twelve outbreaks were identified through environmental surveillance, which can be explained by the large number of environmental surveillance sites in Nigeria, which accounts alone for 32% of all of the sites in the African region (n=199/616) [30]. In addition, although there has been a large expansion of environmental surveillance by GPEI across the last decade as a supplementary surveillance mechanism - it cannot be conducted in rural areas with unconverging sewage systems [30,39]. In early phases of outbreak response, testing healthy children from affected communities has also been used to quantify outbreak size quickly. The relative value of enhanced contact sampling with respect to environmental surveillance and healthy child testing sensitivity in a given population requires further investigation.”

Also, on line 457:

“Its clear value has been established here in one of the key countries where cVDPV2 transmission is proving difficult to stop.”

Was replaced by:

“While its optimal timing of implementation has now established in Nigeria, where VDPV2 transmission is proving difficult to stop, its cost-effectiveness alongside environmental surveillance require further investigation across multiple settings.”

Comment 3.13

**I agree that contact sampling, if done, should be done to maximise its utility. But I would like to see an elaboration of what this might mean. Could you define utility here? Especially at a time where GPEI has to slash its budget by ~40% it’s difficult to know if investing in more contact sampling is a good use of resource. It was commented that contact sampling has been used in specific outbreaks (with known issues in surveillance), so perhaps this advice could be updated but it’s unrealistic to implement everywhere. What steps would be needed to establish the ‘middle ground’?**

Authors’ reply

We agree with the reviewer that contact sampling is costly and time-consuming. Our paper didn’t aim to advise on conducting contact sampling systematically, and we have now revised our language with respect to the comments above. We utilise the opportunity provided by the systematic sampling conducted in Nigeria to gain insights into how contact sampling should be carried out if it is conducted. On the other hand, contact sampling is frequently employed by the program. Our results help determine when contact sampling should be collected to identify false negatives and when it would not bring additional value (i.e. when AFP case stool samples were collected within 5 days). In our dataset, 35% of the AFP cases were collected within 5 days. Excluding them from contact sampling could help reduce the workload associated with contact sampling and thus save money and time, but again, in a context where contact sampling need to be conducted.

We added line 428:

“In the context of necessary cost-reduction measures, limiting contact sampling to AFP cases who haven’t had their stool collected within 5 days can help alleviate lab workload and associated expenses when contact sampling is deployed, e.g., during outbreak response. In this study, 35% of the AFP had their stool collected within 5 days. Excluding them from contact sampling would represent non-negligible savings.”

Comment 3.14

**Environmental surveillance has not been mentioned in this analysis, or discussion. ES would have been carried out alongside, and perhaps a comparison was outside the scope of analysis. But I think ES and its dual role should be mentioned in the Discussion at least.**

Authors’ reply

We agree with the reviewer that environmental surveillance should be mentioned. We added a comparison of their utility in the introduction and added a sentence in the discussion as well as the S2 Table, which describes emergence groups and the surveillance type used to obtain the first 10 detections.

We added line 84:

“Contact sampling is an important surveillance tool for the GPEI, as it can be deployed quickly whenever and wherever outbreaks happen. It complements environmental surveillance, which relies on longitudinal wastewater testing, but only is applicable to large portions of populations who are connected to a convergent sewage network. Extended and systematic contact sampling has rarely been done due to the increased cost and laboratory workload, data on real-world polio contacts are scarce, and studies on the best strategies to implement it are non-existent. ”

On line 433, we already had:

“Contact sampling and environmental surveillance are complementary surveillance strategies to increase surveillance sensitivity. New molecular tools could help reduce diagnostic processing time and increase testing capacity, allowing for an increased scale of contact sampling [38]. Between 2015 and 2023, AFP contact sampling only contributed to the first 10 detections of VDPV2 outbreaks in three out of twelve outbreaks, although most outbreaks emerged outside the period of enhanced AFP contact sampling. Further work can investigate how this surveillance method contributes to defining the scope of response and understanding of spatial spread. However, eight out of the twelve outbreaks were identified through environmental surveillance, which can be explained by the large number of environmental surveillance sites in Nigeria, which accounts alone for 32% of all of the sites in the African region (n=199/616) [30]. In addition, although there has been a large expansion of environmental surveillance by GPEI across the last decade as a supplementary surveillance mechanism - it cannot be conducted in rural areas with unconverging sewage systems [30,39]. In early phases of outbreak response, testing healthy children from affected communities has also been used to quantify outbreak size quickly. The relative value of enhanced contact sampling with respect to environmental surveillance and healthy child testing sensitivity in a given population requires further investigation.“

*S2 Table: Surveillance system provenance of the first 10 detections for each emergence group that has affected Nigeria between 2015 and 2023. The surveillance type refers to the surveillance system through which the virus was detected*

| *Emergence group* | *year of first detection* | *Total number of detections* | *surveillance type for the first 10 detections* | | | |
| --- | --- | --- | --- | --- | --- | --- |
|  |  |  | *AFP* | *contact* | *ES* | *other* |
| *NIE-SOS-2* | *2016* | *2* | *1* | *1* | *0* | *0* |
| *NIE-JIS-1* | *2018* | *265* | *3* | *0* | *7* | *0* |
| *NIE-SOS-3* | *2018* | *26* | *0* | *0* | *10* | *0* |
| *NIE-SOS-7* | *2019* | *58* | *3* | *3* | *4* | *0* |
| *NIE-KGS-1* | *2019* | *8* | *3* | *0* | *4* | *1* |
| *NIE-SOS-6* | *2019* | *5* | *0* | *0* | *5* | *0* |
| *NIE-KGS-2* | *2019* | *5* | *2* | *0* | *0* | *3* |
| *NIE-SOS-5* | *2019* | *2* | *1* | *0* | *0* | *1* |
| *NIE-SOS-4* | *2019* | *4* | *0* | *0* | *4* | *0* |
| *NIE-ZAS-1* | *2020* | *1112* | *6* | *0* | *4* | *0* |
| *NIE-SOS-8* | *2020* | *11* | *2* | *7* | *1* | *0* |
| *NIE-KBS-1* | *2021* | *3* | *3* | *0* | *0* | *0* |

Comment 3.15

**“The rapid detection of cVDPV2 emergence and circulation is critical to stop transmission as quick interventions are easier in limited geographical units.” Rephrase, and would be better to reference evidence that faster responses result in smaller outbreaks.**

Authors’ reply

We agree with the reviewer that this sentence was unclear.We have rephrased and added a reference to the paper from Shaw *et. al.*, ”Time Taken to Detect and Respond to Polio Outbreaks in Africa and the Potential Impact of Direct Molecular Detection and Nanopore Sequencing”.

On line 52:

The rapid detection of cVDPV2 emergence and circulation is critical to stop transmission as quick interventions are easier in limited geographical units.

Was replaced by:

“The rapid detection of cVDPV2 emergence and circulation is critical to stop transmission, as quick interventions result in smaller outbreaks [4].”

Comment 3.16

**In a few places, it feels like unnecessary use of “the” before a noun, but this is coming from a native English speaker, so it could be my grammar that is at fault.**

Authors’ reply

We thank the reviewer for their comment; the manuscript has been reviewed for grammar thoroughly.

Comment 3.17

**“New direct detection methods produce results faster with a higher throughput of samples and could increase the testing of asymptomatic individuals.” It’s not strictly the direct detection methods that increase testing, but their availability could increase the potential for testing asymptomatic individuals.**

Authors’ reply

We thank the reviewer for pointing out this mistake.

On line 60:

“New direct detection methods produce results faster with a higher throughput of samples and could increase the testing of asymptomatic individuals.”

Was replaced by:

“New direct detection methods yield faster results and higher sample throughput, potentially increasing testing capacity for asymptomatic individuals.”

Comment 3.18

**Suitable reference for POLIS.**

Authors’ reply

We thank the reviewer for pointing out this mistake.

The POLIS reference:

“About POLIS n.d. https://extranet.who.int/polis/About (accessed June 27, 2024).”

Has been replaced by:

“WHO. Polio Information System n.d. https://extranet.who.int/polis/public/CaseCount.aspx (accessed June 27, 2024).”

Comment 3.19

**Need to explain why not all VDPV2 are cVDPV2**

Authors’ reply

We thank the reviewer for their suggestion and have added on line 142:

“Most of the VDPV2 cases identified in Nigeria have been defined as ‘circulating’ (referred to as cVDPV2), where that has been genetic evidence or person-to-person evidence in the community. However, there are also cases classified as iVDPV2, which occur when individuals with immunodeficiency disorders excrete VDPV2, and ambiguous VDPV2 (aVDPV2), which are isolates either from environmental samples without evidence of circulation or from single individuals without a known immunodeficiency.”

Comment 3.20

**Table 1, Please add column for VDPV2 positive AND contact data available. Presumably this sums to n=331?**

Authors’ reply

We’ve added a column to the table with the VDPV2 positive AFP having contact data. This is not summing up to 331 because we included the false negative in the sum of this column. A note has been added under the table to clarify.

Line 223, Table 1:

*Table 1: Distribution of the number of AFP cases and their contacts in Nigeria with stool samples collected between the 1st January 2017 and the 30th November 2023.*

| *Year of stool collection* | *Number of AFP cases* | *Number of AFP with contacts* | *Number of contacts collected* | *Number of VDPV2 positive AFP1* | *Number of VDPV2 positive contacts* |
| --- | --- | --- | --- | --- | --- |
| *2017* | *16,762* | *1,966* | *5,555* | *0* | *0* |
| *2018* | *9,767* | *1,643* | *4,655* | *34* | *5* |
| *2019* | *7,645* | *1,488* | *4,259* | *25* | *5* |
| *2020* | *6,447* | *1,141* | *3,228* | *8* | *8* |
| *2021* | *7,955* | *3,908* | *11,350* | *425* | *312* |
| *2022* | *10,491* | *4,026* | *11,303* | *46* | *31* |
| *2023* | *11,333* | *3,745* | *10,750* | *62* | *41* |

*1 data extracted from POLIS on the 8th of January 2024*

Was replaced with:

“Table 1: Distribution of the number of AFP cases and their contacts in Nigeria with stool samples collected between the 1^st^ January 2017 and the 30^th^ November 2023.

| **Year of stool collection** | **Number of AFP cases^1^** | **Number of AFP with contacts** | **Number of contacts collected** | **Number of VDPV2 positive AFP^1,2^** | **Number of VDPV2 positive AFP^1,2^ having contacts** | **Number of VDPV2 positive contacts** |
| --- | --- | --- | --- | --- | --- | --- |
| 2017 | 16,762 | 1,966 | 5,555 | 0 | 0 | 0 |
| 2018 | 9,767 | 1,643 | 4,655 | 34 | 15 | 5 |
| 2019 | 7,645 | 1,488 | 4,259 | 25 | 10 | 5 |
| 2020 | 6,447 | 1,141 | 3,228 | 8 | 3 | 8 |
| 2021 | 7,955 | 3,908 | 11,350 | 425 | 339 | 312 |
| 2022 | 10,491 | 4,026 | 11,303 | 46 | 37 | 31 |
| 2023 | 11,333 | 3,745 | 10,750 | 62 | 54 | 41 |
| **TOTAL** | **70,400** | **17,917** | **51,100** | **600** | **458** | **402** |

^1^ data extracted from POLIS on the 8^th^ of January 2024

^2^ including true positive VDPV2 AFP (total n= 477) and false negative VDPV2 AFP (total n=123)”

Comment 3.21

**Factors associated with VDPV2 contact, please re-phrase the last sentence, it is not clear what a ‘local epidemic’ is**

Authors’ reply

We agree with the reviewer that using this term was confusing, and we use the same term “number of true positive AFP in the state and month before” throughout the text.

On line 272,

“The factors associated with a higher chance of finding a VDPV2 contact were the delay between paralysis onset and the contacts’ stool collection (with a decrease of 40% in the probability of finding VDPV2-positive contacts between 0 and 15 days after onset); the age of the AFP (with a higher mean probability for younger children with AFP (0.51 (sd=0.19) between 12 and 36 months versus 0.41 (sd=0.18) between 37 and 48 months old)); and, the local epidemic level which also increased the probability of VDPV2-positive contacts when the number of VDPV2 AFP in the state increased (Fig 2).”

Was replaced by:

“The factors associated with a higher chance of finding a VDPV2 contact were the delay between paralysis onset and the contacts’ stool collection (with a decrease of 40% in the probability of finding VDPV2-positive contacts between 0 and 15 days after onset); the age of the AFP (with a higher mean probability for younger children with AFP (0.51 (sd=0.19) between 12 and 36 months versus 0.41 (sd=0.18) between 37 and 48 months old)); and, the number of true positive AFP detected in the same State within a month which also increased the probability of VDPV2-positive contacts when the number of VDPV2 AFP in the state increased (Fig 2).”

Comment 3.22

**The reference to the Somalia data is interesting. You have a censored (up to 3) distribution in your data, and I wonder how the data are presented in Somalia to know if the distribution is similar? Having 5 contacts would be even harder/expensive to implement though!**

Authors’ reply

The paper from Moturi et al. is very interesting. The distribution of the contacts in their study is stated as follows in their paper.

“Among the 546 AFP cases, 41 (7.5%) had a single contact tested, 49 (9.0%) had 2 contacts, 225 (41.2%) had 3 contacts, 31 (5.7%) had 4 contacts, 56 (10.3%) had 5 contacts, 16 (2.9%) had 6 or more contacts, and 128 (23.4%) had no contact stool specimens.”

In our study, 75% of the AFP had no contact stool specimens (53085/71002), 0.8% had 1 contact (586/71002), 2% had 2 contacts (1589 /71002), 22% had 3 contacts (15732/71002), and 0.01% had more than 3 contacts (10/71002). We didn’t have much information in our study to understand the impact of having a different number of contacts, as most of the AFP having contact sampled would have 3. However, Their sample size is much smaller and they didn’t look at the interval between onset and stool collection.
