# Supplementary material for: Quantifying the role of contact sampling for poliovirus detection in Nigeria
Source: PLOS Glob Public Health. 2026 May 13;6(5):e0006371. doi: 10.1371/journal.pgph.0006371 (PMC13170847; doi:10.1371/journal.pgph.0006371)
Supplement: Attachment — Submitted filename: Reply to reviewer comments _v2.docx [file pgph.0006371.s019.docx]

Reply to reviewer comments

PGPH-D-25-01818

Title: Quantifying the role of contact sampling for poliovirus detection in Nigeria

Dear Editor,

Thank you for letting us revise our manuscript further. We have addressed the 4th reviewer's comments. In particular, we added more methodological details about the machine learning models in the supplementary material and have included a discussion on model generalisation in the main text.

The line numbers provided in this document refer to the paper submitted with the track changes.

Reviewer #4:

Comment 4.1

**there is overfitting concern for one of the models but that is something the authors acknowledge - I am surprised by the difference in training and testing, however, considering cross-validation was used**

Authors’ reply

We thank the reviewer for their comment and agree that even when using cross-validation, the training and fitting set gave different levels of classification error. This might be caused by the relatively small sample size. We note in the discussion that overfitting would be reduce if more data become available from other contexts (lines 383-384).

Comment 4.2

**an established research checklist should be used, to provide clear information on key aspects e.g. how missing data were dealt with. Some information on missing data is provided on one of the responses, but that needs to be clearly stated in the paper, in accordance with STROBE or other for example.**

Authors’ reply

We thank the reviewer for their comment and have added information on data cleaning and missing data in two supplementary tables (S1 Table, S2 Table). We have also added some details in the method part of the main text.

We added line 142:

“AFP data were checked for missing or erroneous data, and records corresponding to non-AFP identifiers (contacts or healthy children) were removed (S1 Table). Contact data were cleaned to correct misspelt dates or locations. Inconsistent dates between paralysis onset and stool collection were recoded as NAs (see S1 Table for further details). Contacts that could not be matched with an index AFP or contained inconsistent dates were removed (S2 Table). ”

In the supplements, we added:

**S1 Table: AFP data cleaning process (data from Nigeria 1st Dec 2016 - 30th Nov 2023). The observations containing errors have either been fully removed from the dataset or the error has been recoded as NAs. In those instances, if the variable with missing information is used in a model, this observation will be excluded in the analysis.**

| Error tested | Nb errors | Action to correct | Nb corrected | Nb remain |
| --- | --- | --- | --- | --- |
| Non-AFP identifier | 6 | Remove observations | 6 | 0 |
| Wrong surveillance type | 0 | Remove observations | 0 | 0 |
| Missing date of onset | 0 | Remove observations | 0 | 0 |
| Results pending and onset date> 6 months | 48 | NA | 0 | 48 |
| Missing date stool 1 and onset date> 6 months | 2 | NA | 0 | 2 |
| Missing date stool 2 and onset date> 6 months | 5 | NA | 0 | 5 |
| Stool collection before onset | 0 | NA | 0 | 0 |
| Stool1 collected more than 6 months after onset | 2 | NA | 0 | 2 |

And:

**S2 Table: Contact data cleaning flowchart (Nigeria 1^st^ Jan 2017 - 30^th^ Nov 2023).** The observations containing errors have been either fully removed from the dataset, corrected based on other contacts (there are 3 contacts sampled per AFP cases in general), or recoded as NAs. In those instances, if the variable with missing information is used in a model, this observation will be excluded.

| Error tested | Nb errors | Action to correct | Nb corrected | Nb remain |
| --- | --- | --- | --- | --- |
| Duplicates | 203 | Remove observations | 203 | 0 |
| Non-contact identifier | 38 | Correct misspellings when possible | 9 | 29 |
| Contact not matched with an AFP based on identifiers | 166 | Correct misspellings when possible | 81 | 86 |
| Date of stool collection <2016 | 8 | Correct the date based on other contacts | 8 | 0 |
| Stools sent to the lab before stool collection | 4 | Correct the date based on other contacts | 4 | 0 |
| Stools sent to the lab more than 20 days after stool collection | 209 | Correct the date based on other contacts | 109 | 100 |
| Stools received by the lab before being sent | 0 | Correct the date based on other contacts | 0 | 0 |
| Samples that need more than 5 days to travel | 860 | NA | 0 | 860 |
| Samples received by the lab more than 300 days after being sent | 2 | Correct the date based on other contacts | 2 | 0 |
| Missing date of the onset of the index APF | 86 | NA | 0 | 86 |
| Samples collected before the onset of the index AFP | 178 | Correct the date based on other contacts if possible | 164 | 14 |

Comment 4.3

**more about the missing data, clear infromation needs to be provided on all variables with missing data and levels, assumed missingness mechanisms, if a complete case analysis was used and why not multiple imputation (even as sensitivity).**

Authors’ reply

Thank you for highlighting more details are required on missing data. We have added more detail on how the datasets have been cleaned in supplementary tables 1 and 2.

Regarding the handling of missing data, we decided to remove records with missing data for the analyses. This decision is based on the small sample size, which limits the chance of imputing information without creating a bias, and also based on the fact that we are interested in the effect of the days of collection for both the AFP and the contacts and the difference between those collection dates. This difference is often very small (<3 days) and generally <7 days. Using imputation could significantly affect our results.

In the S1 Text, we added:

“Observations with missing data on any factor will be ignored.”

Comment 4.4

**why did the model need to be simplified by removing sex and time between onset? did the authors try to include these variables and examine if the inclusion of these did not improve performance? a clear justification is needed on modelling choices.**

Authors’ reply

For each model, we selected the one that gave the best performance. The variables that did not improve the model's performance have been removed. We clarified this in the text.

We added line 177:

“For each model, the set of factors providing the best-fitting yet parsimonious model has been chosen.”

Also, in S1 Text:

“The factors selected to build the ensemble model include the same factors as the VDPV2 contact model except for the sex of the AFP case and the time between the onset of AFP and the contacts’ stool collection. Those factors have been removed to simplify the model and improve the model’s performance. Also, the year has been added because the definition of the FN AFP and the testing recommendations have changed over time (Table S1).”

Comment 4.5

**the code availability on github is certainly a step in the right direction, towards replicability. it would also be useful to summarise the hyperparameters in a table for easy access.**

Authors’ reply

We agree with the reviewer and have added a table in the supplementary information summarising the hyperparameters tested to tune the model.

We added in S1 Text:

“Table a.: model hyperparameters and ranges tested through nested resampling.

| **Hyperparameter** | **Min value** | **Max value** | **Evaluation method** | **N evaluations** |
| --- | --- | --- | --- | --- |
| number of trees | 50 | 3000 | grid | 15 |
| interaction depth | 1 | 5 | grid | 15 |

”

Comment 4.6

**report calibration metrics as well, don't focus on discrimination only. is there a particular rationale for the thresholds used? Youden? or utility cost? Why aren't ROC curves reported?**

Authors’ reply

We thank the reviewer for their comment. As suggested, we have now added the ROC curves to the supplement of the paper. We also added a table summarising the performances of the models. We used a probability threshold of 0.5, which is the optimal threshold for the contact model. However, the best threshold for the false negative model is 0.25. Despite this difference, we chose to maintain the same threshold for both models in order to interpret them together effectively. Additionally, it is important to ensure that we maintain a good level of specificity in our context.

On line 312, we, added:

“The ROC curves for this model set the best at 0.25. Despite this difference, we chose to maintain the same threshold (0.5) for both models in order to interpret them together effectively (S4 Fig and S7 Fig). “

In supplement, we added:

“

S4 Fig: ROC curves for the model of the probability of VDPV2 contact. The 10 curves represented correspond to the first 10 iterations of the model.

S7 Fig: ROC curves for the model of the probability of FN AFP. The 10 curves represented correspond to the 10 iterations of the model.

*S6 Table: performances of the models averaged across model iterations and overall when using median predictions of each ensemble model*

| *model* | *probability of finding a cVDPV2 contact* | *probability of false negative AFP* |
| --- | --- | --- |
| *performances averaged across model iterations:* |  |  |
| *fitting-set median classification error (IQ)* | *0.45 (IQ=0.43-0.48)* | *0.26 (0.25-0.28)* |
| *cross-validation set median classification error (IQ)* | *0.46 (IQ=0.44-0.49)* | *0.40 (0.40-0.50)* |
| *mean sensitivity (sd)* | *0.74 (0.04)* | *0.36 (0.26)* |
| *mean specificity (sd)* | *0.66 (0.06)* | *0.98 (0.01)* |
| *mean accuracy (sd)* | *0.69 (0.04)* | *0.83 (0.06)* |
|  |  |  |
| *overall ensemble model performance:* |  |  |
| *sensitivity* | *0.81* | *0.3* |
| *specificity* | *0.71* | *0.99* |
| *accuracy* | *0.75* | *0.82* |

”

Comment 4.7

**if i read this correctly, the AFP definition changes after 2021. that means a sensitivity analysis may be needed, only looking at post 2021 data.**

Authors’ reply

We thank the reviewer for their comment. This is a very interesting point. However, there were no FN AFP before 2021 in our data (as shown in supplementary figure 5 (below)). We looked into different approaches we could use to account for this problem and concluded that including the year of paralysis onset in the model is the simplest way to account for this bias. We added a point in the discussion to emphasise this bias.

S5 Fig:

*S5 Fig: Timeliness of the contact stool collection for the FN AFP. a. distribution of the delays to collect the VDPV2 AFP ‘s contacts’ stool samples depending if they are of a True Positive (TP) AFP in grey or a False Negative (FN) AFP in gold; b. temporal distribution of the FN AFP depending if their contacts’ stools have been collected late (more than 30 days after the onset) in pink, between 14 and 30 days after the onset in orange or timely in bleu; spatial distribution of the FN AFP depending if their contacts’ stools have been collected late (more than 30 days after the onset) in pink, between 14 and 30 days after the onset in orange or timely in bleu.*

We added line 390:

“Fourthly, the definition of a false negative AFP case changed in 2021. Before 2021, AFP cases with negative stool samples were considered negative for poliovirus as long as the stool samples were adequate; having a VDPV2-positive contact did not change their case classification. Since 2021, all AFP cases with a VDPV2-positive contact have been classified as VDPV2-positive, regardless of their own stool results. This limits our understanding of the false negative VDPV2 AFP cases prior to that date. However, the new definition is still in use, so our analysis reflects the current situation.. ”

Comment 4.8

**generalisability needs to be discussed as a limitation in the relevant section, explicitly.**

Authors’ reply

We agree with the reviewer and have added a section in the discussion.

We added line 399:

“The generalisability of our results is supported by the standardised definitions, laboratory methods and data collection guidelines used globally by the polio surveillance program. However, each country has its own socio-cultural context and surveillance challenges that might differ from those in Nigeria.”

Comment 4.9

**summarise performance metrics in an accessible table, there isn't one at the minute.**

Authors’ reply

We thank the reviewer for their comment and have added the S6 Table to summarise the models’ performances.

*S6 Table: performances of the models averaged across model iterations and overall when using median predictions of each ensemble model*

| model | probability of finding a cVDPV2 contact | probability of false negative AFP |
| --- | --- | --- |
| *performances averaged across model iterations:* |  |  |
| fitting-set median classification error (IQ) | 0.45 (IQ=0.43-0.48) | 0.26 (0.25-0.28) |
| cross-validation set median classification error (IQ) | 0.46 (IQ=0.44-0.49) | 0.40 (0.40-0.50) |
| mean sensitivity (sd) | 0.74 (0.04) | 0.36 (0.26) |
| mean specificity (sd) | 0.66 (0.06) | 0.98 (0.01) |
| mean accuracy (sd) | 0.69 (0.04) | 0.83 (0.06) |
|  |  |  |
| *overall ensemble model performance:* |  |  |
| sensitivity | 0.81 | 0.3 |
| specificity | 0.71 | 0.99 |
| accuracy | 0.75 | 0.82 |

Comment 4.10

**I would recommend structuring the abstract for clarity and balance**

Authors’ reply

We thanks the reviewer for their comment and have edited our abstract to improve clarity.

On line 14:

*“**Sensitive poliovirus surveillance is critical during the polio endgame and relies on systematic testing of stool samples from reported acute flaccid paralysis (AFP) cases. Collecting stool samples from healthy close contacts of AFP cases, preferably contacts aged under 5 years, is a potential method for enhancing surveillance. The introduction of type 2 novel oral polio vaccine (nOPV2) in Nigeria from 2021 under* *Emergency Use Listing was accompanied by an unprecedented intensification of contact sampling, but it is unclear how this affected surveillance sensitivity**.*

*Here, we analysed data from 71,002 AFP cases and 51,100 associated contacts sampled in Nigeria between January 2017 and November 2023 to quantify the added value of contact sampling and assess how it can be optimised. A total of 600 serotype-2 vaccine-derived poliovirus AFP cases were reported, of which 123 (20.5%) were identified through the presence of the virus in the stool of their contacts (‘false negative’ cases). Boosted Regression Trees ensemble models were used to identify the factors associated with the probability of a type-2 vaccine-derived poliovirus positive contact and for the AFP case to be a false negative.*

*AFP cases and contacts had a higher chance of concordant type-2 vaccine-derived poliovirus test results when stools were collected within 5 days of paralysis onset, whilst false negative AFP cases were most likely 5-15 days after paralysis onset.*

Our results suggest that contact sampling enhances the sensitivity of poliovirus surveillance, especially for AFP cases with inadequate stool samples or with stool samples collected 5-15 days after the onset of paralysis. ”

Comment 4.11

**can the authors provide clearer definitions of the fitting test / inner test and cross-validation test sets?**

Authors’ reply

We agree with the reviewer that the clarity of this section could be improved, thank you for this comment. To clarify, the fitting dataset is the randomly selected data used to build the model and estimate model parameters. The cross-validation dataset is only used after the model is fitted, and is then used to validate and assess the model's performance. When building a BRT model, the fitting set is divided into several sets to train and test the model at each step, which is what we call the inner-tests. We added clarification in the supplementary material.

In S1 Text:

“We identified factors associated with the probability of finding i) a VDPV2 contact, or ii) a False Negative AFP through fitting Boosted Regression Trees (BRT). This machine learning approach allows for non-linear relationships of variables in the model and maximises the explanation of the variance by each factor [1]. At each step of the tree-building process (i.e. each node), the model chooses a factor’s threshold that best discriminates the data into two groups and thus maximises the explanation of the variance. Then, at the next step/node, the model will only try to address the residual variance and so on until the stopping criteria are reached. Figure S1 presents the flow diagram of the ensemble models built for this analysis. The ensemble models were built by iterating through a series of BRT models. At each iteration, the data is resampled and divided into two sets: the fitting set, used to tune the hyperparameters, fit and inner-test the model; and, the cross-validation (CV) set, used to test the performance of the model on data that haven’t been used to fit the model. Based on the fitting set, a BRT model is built, the factors’ importance is assessed, random and spatial resampling are realised to inner-test the performance of the model and, predictions are calculated on the full data set and simulated data to obtain the marginal effects. The predictions calculated on the CV set are then used to assess the model's accuracy.”

Was replaced by:

“We identified factors associated with the probability of finding i) a VDPV2 contact, or ii) a False Negative AFP through fitting Boosted Regression Trees (BRT). This machine learning approach allows for non-linear relationships of variables in the model and maximises the explanation of the variance by each factor [1].

How individual BRT models are built:

At each step of the tree-building process (i.e. each node), the model chooses a factor’s threshold that best discriminates the data into two groups and thus maximises the explanation of the variance. Then, at the next step/node, the model will only try to address the residual variance and so on until the stopping criteria are reached. To select the best combinations at each step, the model divides the data into training and test sets, repeatedly resampling and testing the model. We refer to this repeated train-and-test process used to build the model as inner testing to better differentiate it from the testing process used across several models to build the ensemble model. Figure S1 presents the flow diagram of the ensemble models built for this analysis.

How the ensemble model is built:

The ensemble model was built by iterating through a series of BRT models. At each iteration, the data is resampled and divided into two sets:

-	the fitting set, used to tune the hyperparameters, fit and inner-test the model; and,

-	-the cross-validation (CV) set, used to test the performance of the model on data that haven’t been used to fit the model.

For each individual fitting dataset, a BRT model is built, the factors’ importance is assessed, random and spatial resampling are realised to inner-test the performance of the model. Predictions are then calculated on the full data set and simulated data to obtain the marginal effects. Finally, the model accuracy is assessed through comparing model predictions and observations using the CV dataset.”

Comment 4.12

**there are undefined acronyms in the abstract**

Authors’ reply

We agree with the reviewer that many acronyms are used in the abstract and throughout the paper. We made sure to define all acronyms when they first appeared and included a list at the end of the paper to facilitate reading.

On Line 445, we added:

*“****List of acronyms***

*AFP: acute flaccid paralysis*

*aVDPV2: ambiguous vaccine-derived poliovirus caused by serotype 2*

*BRT: Boosted Regression Trees*

*CE: classification error*

*CV: cross-validation*

*cVDPV: circulating vaccine-derived poliovirus*

*cVDPV2: circulating vaccine-derived poliovirus caused by serotype 2*

*EUL: WHO Emergency Use Listing*

*FN: false negative*

*GPEI: Global Polio Eradication Initiative*

*IQ: interquartile*

*nOPV2: type 2 novel oral polio vaccine*

*OPV: oral polio vaccine*

*POLIS: Polio Information System*

*SIA: supplementary immunisation activities*

*VAPP: Vaccine-associated paralytic poliomyelitis*

*VDPV2: vaccine-derived poliovirus type 2*

*WHO: World Health Organization “*
